# Associations between metabolic indices and the prevalence of simple renal cysts among hospitalized patients with hypertension in Xinjiang, China: a large-scale cross-sectional study

**DOI:** 10.3389/fcvm.2026.1911446

**Published:** 2026-08-12

**Authors:** Huimin Ma, Qing Zhu, Xintian Cai, Delian Zhang, Jing Hong, Luo Qin, Nanfang Li

**Affiliations:** 1 Graduate School, Xinjiang Medical University, Urumqi, Xinjiang, China; 2 Hypertension Center of People's Hospital of Xinjiang Uygur Autonomous Region, Xinjiang Hypertension Institute, NHC Key Laboratory of Hypertension Clinical Research, Key Laboratory of Xinjiang Uygur Autonomous Region “Hypertension Research Laboratory”, Xinjiang Clinical Medical Research Center for Hypertension (Cardio-Cerebrovascular) Urumqi, Xinjiang, China; 3 Department of Cardiopulmonary Vascular Medicine, Sichuan Provincial People’s Hospital, Chengdu, Sichuan, China

**Keywords:** cross-sectional study, hypertension, metabolic indices, prevalence, simple renal cysts

## Abstract

**Background:**

Metabolic dysfunction may be associated with simple renal cysts (SRCs) in patients with hypertension, but the relationships between routinely available metabolic indices and SRC prevalence remain unclear. We therefore aimed to examine the associations between six routinely available metabolic indices and SRC prevalence among hospitalized patients with hypertension.

**Methods:**

This cross-sectional study included 35,000 adults hospitalized with hypertension between January 1, 2014, and October 31, 2025, who had complete data required to calculate the metabolic indices and available renal ultrasonography or abdominal CT findings. Patients aged <18 years, pregnant patients, and those with major hepatic, renal, endocrine, urinary tract, malignant, or non-simple cystic kidney conditions were excluded. Additional exclusions included prior mineralocorticoid receptor antagonist use and extreme values. Associations of six metabolic indices with prevalent SRCs were examined using multivariable logistic regression, supplemented by RCS, ROC, subgroup, sensitivity, and E-value analyses.

**Results:**

Among 35,000 participants, 7,250 (20.7%) had SRCs. SRC prevalence increased progressively across quartiles of all six metabolic indices (all *P* for trend <0.001). In fully adjusted models, each 1-SD increase in the six indices was associated with higher odds of SRCs, with ORs ranging from 1.19 (95% CI: 1.16–1.22) for TG/HDL-C to 1.65 (95% CI: 1.59–1.71) for METS-VF. Compared with Q1, participants in Q4 had adjusted ORs ranging from 1.48 (95% CI: 1.35–1.61) to 3.76 (95% CI: 3.47–4.09). Similar associations were observed for large and multiple SRCs. RCS analyses showed generally increasing exposure-response relationships, and METS-VF had the highest observed discriminative performance for prevalent SRCs (AUC = 0.654).

**Conclusion:**

Higher levels of all six metabolic indices were independently associated with a greater prevalence of SRCs in patients with hypertension, with consistent associations observed for large and multiple cysts. Among the indices evaluated, METS-VF showed the strongest association and the highest observed discriminative performance, suggesting that visceral adiposity-related metabolic dysfunction may be particularly relevant to SRC burden. Prospective studies are needed to confirm the temporal relationship and clinical utility of these findings.

## Introduction

Simple renal cysts (SRCs) are common cystic renal lesions that typically appear as well-defined, thin-walled, anechoic lesions without septa, nodules, or solid components on imaging (1). With the increasing use of routine abdominal and renal imaging, SRCs are being detected more frequently. Although SRCs are often regarded as benign incidental findings, accumulating evidence suggests that their clinical significance may not be entirely neutral. SRC prevalence increases with age and is higher in men and smokers (2–4), and SRCs have been associated with hypertension, impaired renal function, albuminuria or proteinuria, and cardiometabolic abnormalities (4–7). Several studies have further reported that the association between SRCs and elevated blood pressure is more evident in individuals with multiple, large, or bilateral cysts (8–10). These associations may be partly explained by renal vascular, hemodynamic, or tubular alterations. These observations support the rationale for studying factors associated with SRCs in patients with hypertension.

Previous studies have provided preliminary evidence linking SRCs to metabolic abnormalities. Shen et al. reported that metabolic syndrome was associated with SRCs and that this association was more pronounced among individuals with larger or multiple cysts (7). Tao et al. subsequently found that metabolic syndrome was associated with greater renal cyst size (11). Evidence from patients with type 2 diabetes has also shown that SRCs are associated with impaired renal function, hyperuricemia, and excessive urinary uric acid excretion (12–14). Collectively, these findings suggest that metabolic dysfunction may be relevant not only to the presence of SRCs but also to greater cyst burden. However, previous investigations have mainly focused on metabolic syndrome, individual metabolic abnormalities, or selected patient populations. The associations of comprehensive metabolic indices with SRC prevalence and cyst burden remain insufficiently characterized in patients with hypertension. Routinely available metabolic indices, including TG/HDL-C, TyG, TyG-WC, TyG-BMI, METS-IR, and METS-VF, integrate information on lipid metabolism, glucose metabolism, adiposity, and body fat distribution (15–19). Although these indices have been widely used to assess insulin resistance and cardiometabolic risk, their associations with SRC prevalence have not been systematically compared in patients with hypertension. Evaluating these indices may provide a more comprehensive understanding of the metabolic characteristics associated with SRCs. Accordingly, the primary aim of this study was to examine the associations between six routinely available metabolic indices and the prevalence of SRCs among hospitalized patients with hypertension. Secondary analyses evaluated their associations with large and multiple SRCs and compared their discriminative performance.

## Methods

### Study population

**Figure 1:**
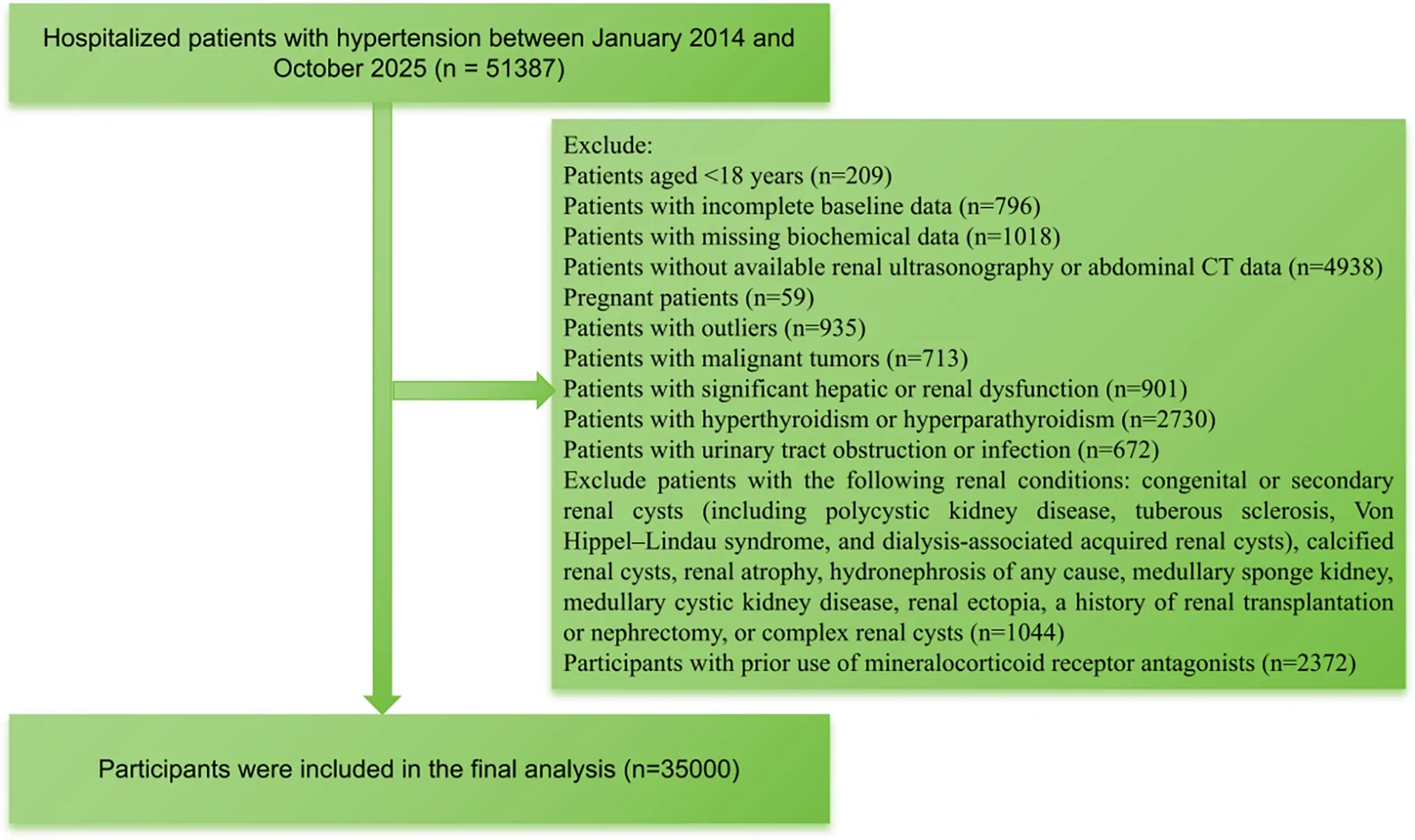
Flowchart for screening of study participants.

This cross-sectional study screened 51,387 patients with hypertension who attended People's Hospital of Xinjiang Uygur Autonomous Region between January 2014 and October 2025. Eligible participants were adults aged ≥18 years with complete data required to calculate the metabolic indices and available renal ultrasonography or abdominal CT data. We excluded patients who were pregnant; had outlying values, malignant tumors, significant hepatic or renal dysfunction, hyperthyroidism or hyperparathyroidism, or urinary tract obstruction or infection; had congenital, secondary, complex, or other major structural renal cystic conditions; or had previously used mineralocorticoid receptor antagonists. After application of these criteria, 35,000 participants were included in the final analysis (Figure 1).

### Data collection and definitions

The six metabolic indices were treated as the exposure variables, whereas SRC status was the primary outcome. Large and multiple SRCs were examined as secondary outcomes. Data were extracted from the hospital electronic health record system and included age, sex, height, weight, body mass index (BMI), blood pressure, waist circumference (WC), current smoking status, current drinking status, medical history, and medication use. Medical history included diabetes mellitus (DM), coronary heart disease (CHD), dyslipidemia, nephrolithiasis, and hyperuricemia. Additional details regarding data collection and variable definitions are provided in the Supplementary Material. Fasting blood samples were obtained after an 8–10-hour overnight fast and analyzed for alanine aminotransferase (ALT), aspartate aminotransferase (AST), serum creatinine (SCr), uric acid (UA), fasting plasma glucose (FPG), total cholesterol (TC), triglycerides (TG), high-density lipoprotein cholesterol (HDL-C), low-density lipoprotein cholesterol (LDL-C), and glycated hemoglobin (HbA1c). Laboratory measurements were performed using a C16000 automated biochemistry analyzer (Abbott Laboratories, Abbott Park, IL, USA) according to standardized laboratory procedures. The metabolic indices were calculated using the following equations:

$$\begin{array}{rl} & \mathrm{TG}/\mathrm{HDL}\text{-}C=\mathrm{TG}(\mathrm{mg}/\mathrm{dL})/\mathrm{HDL}\text{-}C(\mathrm{mg}/\mathrm{dL});\\ & \mathrm{TyG}=\ln[\left(\mathrm{TG}(\mathrm{mg}/\mathrm{dL})\times \mathrm{FPG}\;(\mathrm{mg}/\mathrm{dL}){/}^{2}\right)];\\ & \mathrm{TyG}\text{-}\mathrm{BMI}=\mathrm{TyG}\times \mathrm{BMI};\\ & \mathrm{TyG}\text{-}\mathrm{WC}=\mathrm{TyG}\times \mathrm{WC};\\ & \mathrm{METS}\text{-}\mathrm{IR}=\ln[2\times \mathrm{FPG}(\mathrm{mg}/\mathrm{dL})+\mathrm{TG}(\mathrm{mg}/\mathrm{dL})]\\ & \;\times \mathrm{BMI}(\mathrm{kg}/m^{2})/\ln[\mathrm{HDL}\text{-}C(\mathrm{mg}/\mathrm{dL})];\\ & \mathrm{METS}\text{-}\mathrm{VF}=4.466+0.011\times [\ln(\mathrm{METS}\text{-}\mathrm{IR}){]}^{3}+3.239\\ & \;\times [\ln(\mathrm{WC}/\mathrm{height}){]}^{3}+0.319\times \mathrm{sex}+0.594\times \ln(\mathrm{age}), \end{array}$$

where sex was coded as 1 for men and 0 for women.

### Outcome assessment

SRCs were assessed by certified radiologists using color Doppler ultrasonography (IU22; Philips Healthcare, Andover, MA, USA) or abdominal CT (SOMATOM Definition Flash; Siemens Healthineers, Germany). On ultrasonography, SRCs were identified as round or oval, homogeneous anechoic lesions with a thin smooth wall, clear margins, and posterior acoustic enhancement. On CT, they were characterized by homogeneous water-like attenuation (−5 to +15 Hounsfield units), a thin imperceptible wall, well-defined margins, and no post-contrast enhancement (20). The presence of SRCs was the primary outcome. Large SRCs (>2 cm) and multiple SRCs (≥2 cysts) were secondary outcomes reflecting cyst burden at the index imaging examination rather than longitudinal progression.

### Statistical analysis

Participants were classified according to the presence or absence of SRCs on the index renal imaging examination. Baseline characteristics were compared using the chi-square test for categorical variables, the independent-samples Student's t-test for normally distributed continuous variables, and the Mann–Whitney U test for nonnormally distributed continuous variables. Missing covariate data were handled using multiple imputation with the mice package in R. Multicollinearity was assessed using variance inflation factors, with values <10 indicating no material multicollinearity (Supplementary Table S1). Each metabolic index was evaluated in separate multivariable logistic regression models both as a continuous variable and as a categorical variable based on quartiles. Effect estimates were presented as ORs with corresponding 95% CIs. Potential nonlinear exposure-response relationships were assessed using restricted cubic spline (RCS) models with four knots at the 5th, 35th, 65th, and 95th percentiles. Subgroup analyses were conducted according to sex, age, BMI, current smoking, and current drinking status, with interaction terms used to assess effect modification. ROC curve analysis was performed to compare the ability of different indices to distinguish SRC status. Sensitivity analyses were performed to assess the robustness of the main findings. Finally, E-value analysis was performed to assess the potential influence of unmeasured confounding on the observed associations. All tests were two-sided, and a *P* value <0.05 was considered statistically significant. Further details are available in the Supplementary Material.

## Results

### Clinical characteristics of participants

Among the 35,000 patients with hypertension, 7,250 were diagnosed with SRCs. Compared with patients without SRCs, those with SRCs were older, more frequently male, and had higher WC, height, BMI, and SBP. Patients with SRCs also had higher levels of ALT, AST, SCr, UA, TG, and FPG, together with lower HDL-C and eGFR levels. LDL-C was only slightly lower, whereas TC did not differ significantly between the two groups. The proportions of participants with DM, CHD, dyslipidemia, nephrolithiasis, and hyperuricemia were significantly higher in the SRC group than in the non-SRC group (Table 1).

**Table 1:** Characteristics of the study population.

Variables	Total	Without SRCs	With SRCs	*P*
	*n* = 35,000	*n* = 27,750 (79.3%)	*n* = 7,250 (20.7%)	
Age (years)	51.18 ± 12.39	50.25 ± 12.40	54.71 ± 11.69	<0.001
Sex (male)	20,584 (58.81)	15,438 (55.63)	5,146 (70.98)	<0.001
WC (cm)	96.00 (88.00, 103.00)	95.00 (88.00, 103.00)	98.00 (90.00, 105.00)	<0.001
Height (cm)	168.00 (161.00, 174.00)	168.00 (161.00, 174.00)	169.00 (162.00, 174.00)	0.011
BMI (kg/m^2^)	26.81 (24.45, 29.41)	26.45 (24.17, 29.30)	28.01 (25.78, 30.49)	<0.001
SBP (mmHg)	146.00 (134.00, 160.00)	146.00 (134.00, 159.00)	148.00 (135.00, 161.00)	<0.001
DBP (mmHg)	90.00 (80.00, 99.00)	90.00 (80.00, 99.00)	89.00 (79.00, 99.00)	0.004
Current smoking (%)	11,795 (33.70)	8,780 (31.64)	3,015 (41.59)	<0.001
Current drinking (%)	10,949 (31.28)	8,254 (29.74)	2,695 (37.17)	<0.001
Laboratory tests				
ALT (U/L)	22.00 (15.00, 33.00)	21.11 (15.00, 32.50)	22.60 (16.00, 33.00)	<0.001
AST (U/L)	21.34 ± 11.79	21.23 ± 11.48	21.73 ± 12.91	0.003
eGFR (mL/min/1.73 m^2^)	110.76 ± 20.27	111.00 ± 20.00	109.87 ± 21.24	<0.001
SCr (mg/dL)	0.72 (0.62, 0.84)	0.72 (0.61, 0.83)	0.75 (0.64, 0.86)	<0.001
UA (µmol/L)	344.74 ± 94.03	341.56 ± 93.89	356.89 ± 93.58	<0.001
HDL-C (mg/dL)	41.20 ± 10.32	41.76 ± 10.43	39.05 ± 9.58	<0.001
LDL-C (mg/dL)	107.25 ± 32.89	107.58 ± 32.79	106.00 ± 33.25	<0.001
TC (mg/dL)	174.78 ± 38.17	174.91 ± 37.94	174.29 ± 39.06	0.232
TG (mg/dL)	134.52 (96.47, 192.27)	130.98 (93.81, 186.74)	148.68 (106.20, 216.82)	<0.001
FPG (mg/dL)	92.65 ± 28.13	90.92 ± 25.86	99.23 ± 34.75	<0.001
TG/HDL-C	4.37 ± 3.61	4.16 ± 3.40	5.15 ± 4.22	<0.001
TyG	8.74 ± 0.61	8.69 ± 0.60	8.91 ± 0.65	<0.001
TyG-WC	454.34 ± 86.47	449.48 ± 84.77	472.95 ± 90.30	<0.001
TyG-BMI	129.15 ± 23.10	127.09 ± 22.75	137.02 ± 22.74	<0.001
METS-IR	43.35 ± 8.72	42.54 ± 8.59	46.45 ± 8.56	<0.001
METS-VF	6.56 ± 0.38	6.53 ± 0.38	6.68 ± 0.34	<0.001
Medical history (%)				
DM	5,568 (15.91)	3,862 (13.92)	1,706 (23.53)	<0.001
CHD	3,231 (9.23)	2,347 (8.46)	884 (12.19)	<0.001
Dyslipidemia	6,604 (18.87)	5,018 (18.08)	1,586 (21.88)	<0.001
Nephrolithiasis	3,905 (11.16)	2,876 (10.36)	1,029 (14.19)	<0.001
Hyperuricemia	7,363 (21.04)	5,705 (20.56)	1,658 (22.87)	<0.001
Medications (%)				
Lipid-lowering drugs	4,010 (11.46)	2,983 (10.75)	1,027 (14.17)	<0.001
ACEIs/ARBs	16,150 (46.14)	12,480 (44.97)	3,670 (50.62)	<0.001
Beta-blockers	6,189 (17.68)	4,722 (17.02)	1,467 (20.23)	<0.001
Calcium channel blockers	18,493 (52.84)	14,287 (51.48)	4,206 (58.01)	<0.001
Diuretics	3,765 (10.76)	2,830 (10.20)	935 (12.90)	<0.001
Antidiabetic agents	4,039 (11.54)	2,990 (10.77)	1,049 (14.47)	<0.001

Data are presented as mean ± standard deviation, median (interquartile range), or n (%), as appropriate.

WC, waist circumference; BMI, body mass index; SBP, systolic blood pressure; DBP, diastolic blood pressure; ALT, alanine transaminase; AST, aspartate transaminase; SCr, serum creatinine; eGFR, estimated glomerular filtration rate; TC, total cholesterol; TG, triglyceride; HDL-C, high-density lipoprotein cholesterol; LDL-C, low-density lipoprotein cholesterol; FPG, fasting plasma glucose; UA, uric acid; TyG, triglyceride-glucose; METS-IR, metabolic score for insulin resistance; METS-VF, metabolic score for visceral fat; DM, diabetes mellitus; CHD, coronary heart disease; ARBs, angiotensin receptor blockers; ACEIs, angiotensin-converting enzyme inhibitors.

### Associations of metabolic indices with SRCs

**Figure 2:**
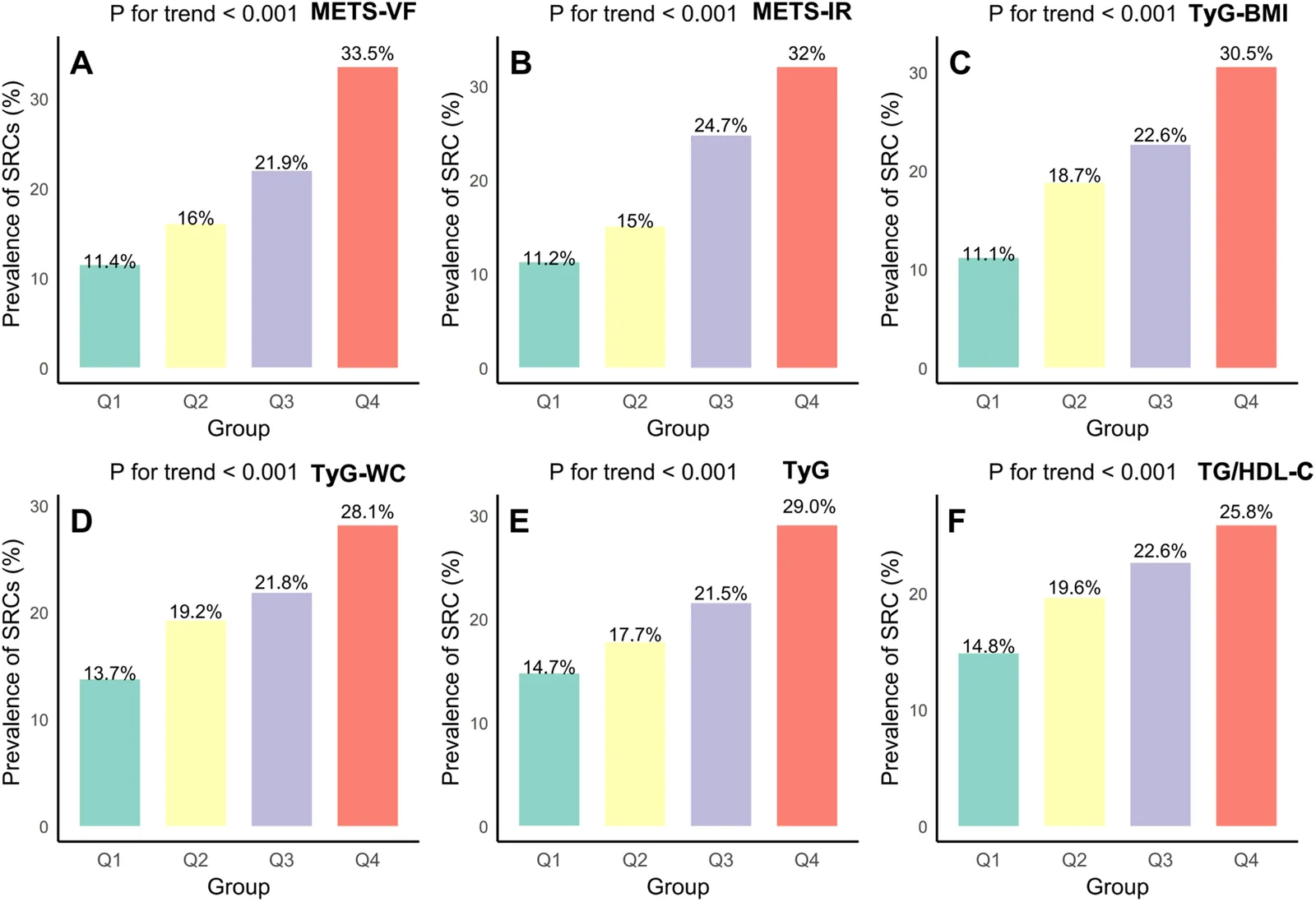
Prevalence of SRCs according to quartiles of six metabolic indices. **(A)** METS-VF; **(B)** METS-IR; **(C)** TyG-BMI; **(D)** TyG-WC; **(E)** TyG; **(F)** TG/HDL-C.

**Figure 3:**
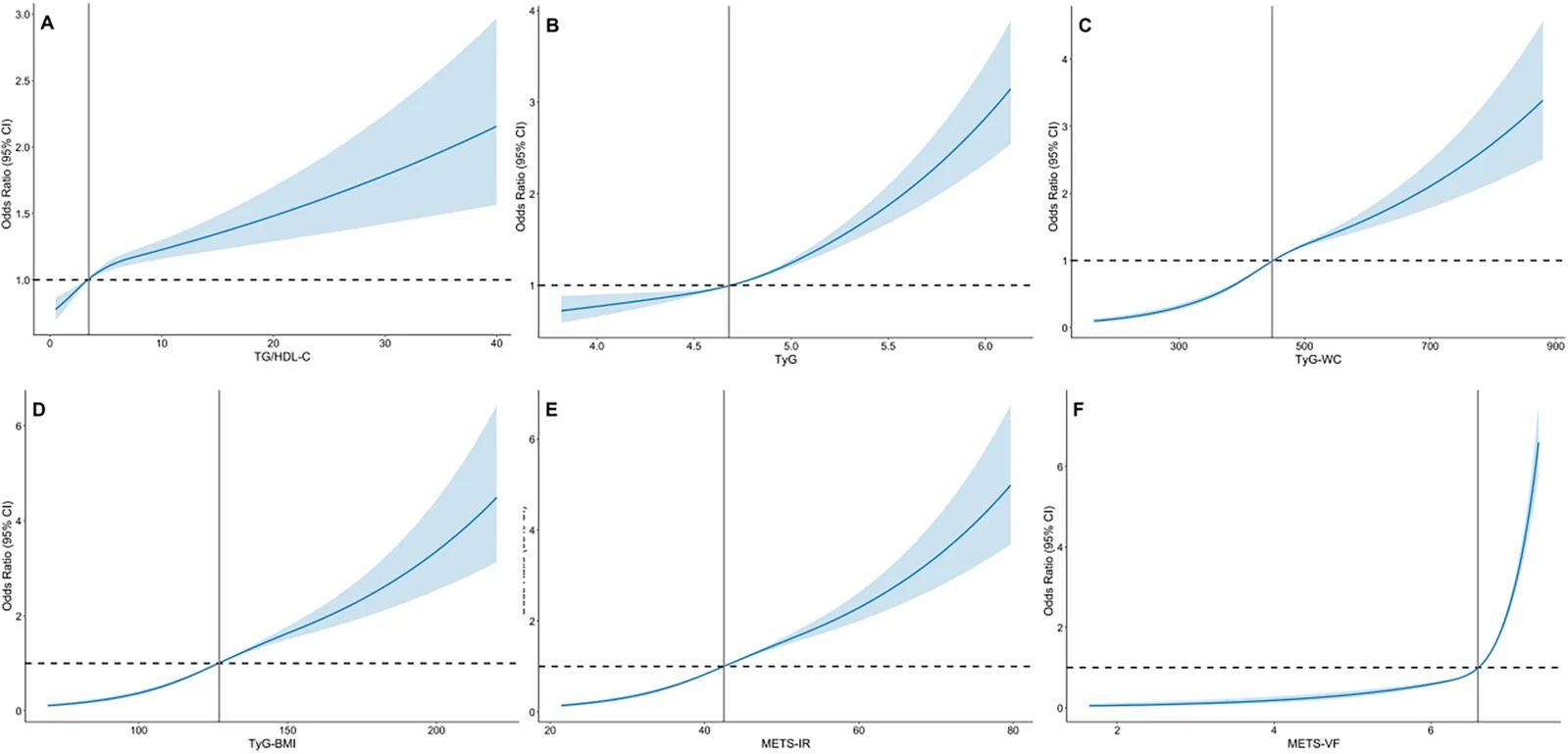
dose-response association between metabolic indices and risk of SRCs. **(A)** TG/HDL-C; **(B)** TyG; **(C)** TyG-WC; **(D)** TyG-BMI; **(E)** METS-IR; **(F)** METS-VF.

Participants were divided into quartile groups for each metabolic index. The prevalence of SRCs rose progressively from the lowest to the highest quartile, and significant linear trends were detected for all six indices (all *P* for trend <0.001; Figure 2). In the fully adjusted model treating these indices as continuous exposures, each 1-SD increase in TG/HDL-C, TyG, TyG-WC, TyG-BMI, METS-IR, and METS-VF corresponded to increased odds of SRCs, with ORs of 1.19 (95% CI: 1.16–1.22), 1.23 (95% CI: 1.19–1.27), 1.35 (95% CI: 1.30–1.40), 1.46 (95% CI: 1.41–1.50), 1.53 (95% CI: 1.49–1.58), and 1.65 (95% CI: 1.59–1.71), respectively (Table 2). When the metabolic indices were analyzed as categorical variables, participants in the highest quartile (Q4) had greater odds of SRCs than those in the lowest quartile (Q1), with adjusted ORs of 1.48 (95% CI: 1.35–1.61), 1.56 (95% CI: 1.43–1.70), 2.09 (95% CI: 1.93–2.27), 3.17 (95% CI: 2.90–3.48), 3.70 (95% CI: 3.38–4.05), and 3.76 (95% CI: 3.47–4.09), respectively (Table 2). RCS analyses showed broadly consistent dose-response patterns, indicating a gradual elevation in SRC odds across the distribution of each metabolic index (Figure 3).

**Table 2:** Associations between metabolic indices and SRCs.

Variables	Model 1	Model 2	Model 3	Model 4
	OR (95% CI)	OR (95% CI)	OR (95% CI)	OR (95% CI)
TG/HDL-C				
Per SD increase	1.26 (1.23–1.29)	1.23 (1.20–1.26)	1.20 (1.17–1.23)	1.19 (1.16–1.22)
Quartiles				
Q1	Reference	Reference	Reference	Reference
Q2	1.40 (1.29–1.51)	1.21 (1.12–1.32)	1.18 (1.09–1.28)	1.18 (1.09–1.29)
Q3	1.68 (1.55–1.82)	1.37 (1.26–1.49)	1.30 (1.20–1.41)	1.30 (1.19–1.41)
Q4	2.00 (1.86–2.16)	1.63 (1.50–1.77)	1.47 (1.35–1.61)	1.48 (1.35–1.61)
*P* for trend	<0.001	<0.001	<0.001	<0.001
TyG				
Per SD increase	1.40 (1.37–1.44)	1.30 (1.27–1.34)	1.26 (1.22–1.30)	1.23 (1.19–1.27)
Quartiles				
Q1	Reference	Reference	Reference	Reference
Q2	1.25 (1.15–1.36)	1.08 (1.00–1.18)	1.04 (0.95–1.13)	1.03 (0.94–1.12)
Q3	1.59 (1.47–1.72)	1.32 (1.21–1.43)	1.22 (1.13–1.33)	1.19 (1.09–1.29)
Q4	2.37 (2.20–2.56)	1.84 (1.70–2.00)	1.66 (1.53–1.81)	1.56 (1.43–1.70)
*P* for trend	<0.001	<0.001	<0.001	<0.001
TyG-WC				
Per SD increase	1.49 (1.44–1.54)	1.42 (1.37–1.48)	1.39 (1.34–1.44)	1.35 (1.30–1.40)
Quartiles				
Q1	Reference	Reference	Reference	Reference
Q2	1.49 (1.38–1.62)	1.46 (1.35–1.59)	1.44 (1.33–1.57)	1.43 (1.32–1.56)
Q3	1.75 (1.62–1.89)	1.64 (1.52–1.79)	1.60 (1.48–1.74)	1.57 (1.45–1.71)
Q4	2.45 (2.27–2.65)	2.29 (2.12–2.48)	2.17 (2.00–2.35)	2.09 (1.93–2.27)
*P* for trend	<0.001	<0.001	<0.001	<0.001
TyG-BMI				
Per SD increase	1.51 (1.48–1.55)	1.53 (1.49–1.57)	1.47 (1.43–1.52)	1.46 (1.41–1.50)
Quartiles				
Q1	Reference	Reference	Reference	Reference
Q2	1.85 (1.70–2.02)	1.73 (1.59–1.89)	1.64 (1.50–1.80)	1.62 (1.48–1.77)
Q3	2.34 (2.15–2.55)	2.25 (2.06–2.46)	2.07 (1.89–2.27)	2.04 (1.86–2.23)
Q4	3.53 (3.26–3.83)	3.63 (3.33–3.95)	3.27 (2.99–3.58)	3.17 (2.90–3.48)
*P* for trend	<0.001	<0.001	<0.001	<0.001
METS-IR				
Per SD increase	1.54 (1.50–1.58)	1.56 (1.52–1.60)	1.55 (1.50–1.59)	1.53 (1.49–1.58)
Quartiles				
Q1	Reference	Reference	Reference	Reference
Q2	1.41 (1.29–1.54)	1.29 (1.18–1.42)	1.29 (1.18–1.41)	1.27 (1.16–1.39)
Q3	2.60 (2.39–2.82)	2.53 (2.32–2.76)	2.53 (2.32–2.76)	2.47 (2.27–2.70)
Q4	3.73 (3.44–4.04)	3.83 (3.51–4.17)	3.82 (3.50–4.17)	3.70 (3.38–4.05)
*P* for trend	<0.001	<0.001	<0.001	<0.001
METS-VF				
Per SD increase	1.71 (1.65–1.77)	1.68 (1.63–1.75)	1.69 (1.63–1.75)	1.65 (1.59–1.71)
Quartiles				
Q1	Reference	Reference	Reference	Reference
Q2	1.49 (1.37–1.63)	1.50 (1.37–1.63)	1.53 (1.40–1.67)	1.54 (1.41–1.68)
Q3	2.19 (2.02–2.38)	2.19 (2.01–2.38)	2.23 (2.05–2.43)	2.21 (2.04–2.41)
Q4	3.94 (3.64–4.26)	3.87 (3.57–4.19)	3.91 (3.60–4.24)	3.76 (3.47–4.09)
*P* for trend	<0.001	<0.001	<0.001	<0.001

Model 1 was unadjusted.

Model 2 was adjusted for age, sex, BMI, current smoking, current drinking, SBP, DBP.

Model 3 was further adjusted for ALT, AST, TG, HDL-C, eGFR, UA, FPG.

Model 4 was additionally adjusted for DM, dyslipidemia, CHD, nephrolithiasis, hyperuricemia, lipid-lowering drugs, antidiabetic agents, diuretics, beta-blockers, calcium channel blockers, and ACEI/ARBs.

WC, waist circumference; BMI, body mass index; TyG, triglyceride-glucose; METS-IR, metabolic score for insulin resistance; METS-VF, metabolic score for visceral fat; OR, odds ratio; CI, confidence interval; Other abbreviations, see Table 1.

### Associations of metabolic indices with large SRCs

The prevalence of large SRCs increased progressively across quartiles of all six metabolic indices, with significant trends observed for each index (all *P* for trend <0.001; Supplementary Figure S1). When the metabolic indices were analyzed as continuous variables, each 1-SD increase in TG/HDL-C, TyG, TyG-WC, TyG-BMI, METS-IR, and METS-VF was associated with higher odds of large SRCs in the fully adjusted model, with ORs of 1.20 (95% CI: 1.15–1.25), 1.27 (95% CI: 1.20–1.35), 1.36 (95% CI: 1.27–1.45), 1.53 (95% CI: 1.46–1.61), 1.61 (95% CI: 1.53–1.69), and 1.79 (95% CI: 1.66–1.94), respectively (Supplementary Table S2). When the metabolic indices were analyzed as categorical variables, participants in Q4 had greater odds of large SRCs than those in Q1, with adjusted ORs of 1.73 (95% CI: 1.47–2.04), 1.75 (95% CI: 1.47–2.07), 2.97 (95% CI: 2.52–3.50), 4.24 (95% CI: 3.53–5.12), 5.84 (95% CI: 4.74–7.22), and 6.38 (95% CI: 5.31–7.71), respectively (Supplementary Table S2). RCS analyses showed similar increasing dose-response patterns for large SRCs across the distributions of these metabolic indices (Supplementary Figure S3).

### Associations of metabolic indices with multiple SRCs

For multiple SRCs, the results were generally in line with those of the primary analysis. The prevalence of multiple SRCs increased stepwise across quartiles for each of the six metabolic indices, with significant trends detected in all comparisons (all *P* for trend <0.001; Supplementary Figure S2). After full adjustment, continuous-variable analyses showed that a 1-SD increment in TG/HDL-C, TyG, TyG-WC, TyG-BMI, METS-IR, and METS-VF corresponded to higher odds of multiple SRCs. The respective ORs were 1.07 (95% CI: 1.02–1.12), 1.10 (95% CI: 1.04–1.17), 1.16 (95% CI: 1.11–1.22), 1.29 (95% CI: 1.12–1.50), 1.58 (95% CI: 1.50–1.66), and 1.67 (95% CI: 1.55–1.80) (Supplementary Table S3). In quartile-based analyses, participants in Q4 had greater odds of multiple SRCs than those in Q1, with adjusted ORs of 1.35 (95% CI: 1.15–1.59), 1.41 (95% CI: 1.19–1.67), 2.86 (95% CI: 2.43–3.37), 3.60 (95% CI: 3.01–4.33), 5.51 (95% CI: 4.50–6.78), and 5.68 (95% CI: 4.73–6.92), respectively (Supplementary Table S3). RCS analyses showed similar increasing dose-response relationships across the distributions of these metabolic indices (Supplementary Figure S4).

### Comparative discriminative performance of the six metabolic indices for prevalent SRCs

**Figure 4:**
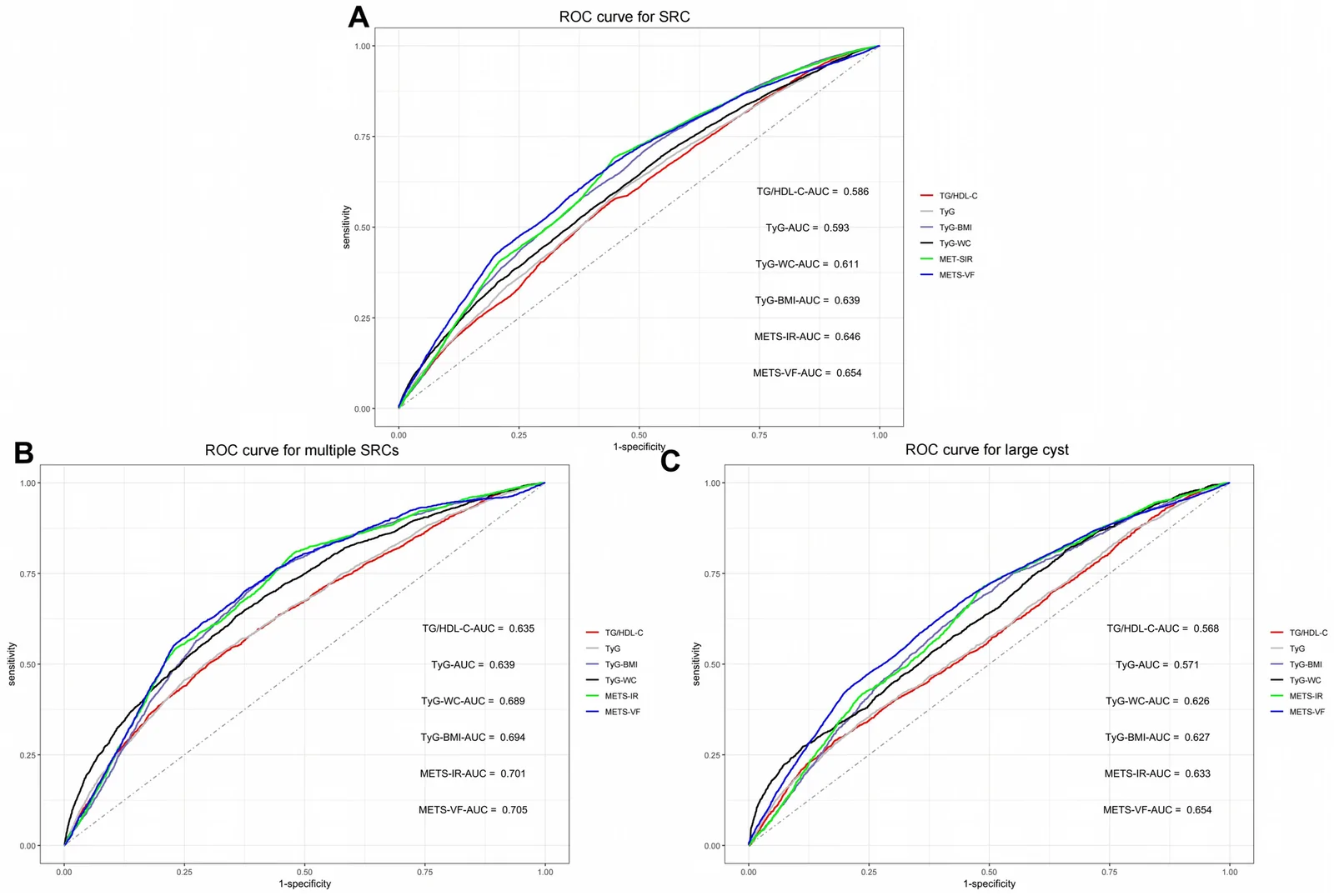
Receiver operating characteristic (ROC) curves assessing the discriminative performance of six metabolic indices for prevalent SRCs. **(A)** SRCs; **(B)** multiple SRCs; **(C)** large SRCs.

ROC curve analysis was performed to compare the discriminative performance of the six metabolic indices for prevalent SRCs and different levels of cyst burden. For prevalent SRCs, METS-VF showed the largest observed AUC (0.654), followed by METS-IR (0.646), TyG-BMI (0.639), TyG-WC (0.611), TyG (0.593), and TG/HDL-C (0.586). For multiple SRCs, the corresponding AUCs were 0.705, 0.701, 0.694, 0.689, 0.639, and 0.635, respectively. For large SRCs, the corresponding AUCs were 0.654, 0.633, 0.627, 0.626, 0.571, and 0.568, respectively (Figure 4).

### Subgroup and sensitivity analysis

To assess the consistency of the regression estimates and potential effect modification, we performed stratified logistic regression analyses according to sex, age, BMI (<24 vs. ≥24 kg/m^2^), smoking status, and drinking status (Table 3 and Supplementary Tables S4, S5). In subgroup analyses, the positive associations were generally consistent across strata. Most interaction tests were not statistically significant. Significant interactions were observed for sex in the TyG-BMI analysis of prevalent SRCs, BMI in the TyG-WC analysis of large SRCs, age in the TyG-BMI analysis of multiple SRCs, current drinking in the METS-VF analysis of prevalent SRCs, and current smoking in the METS-VF analysis of multiple SRCs. Given the number of subgroup comparisons, these findings should be interpreted as exploratory and require confirmation in independent cohorts. Sensitivity analyses excluding participants with obesity, hyperuricemia, nephrolithiasis, diabetes mellitus, or dyslipidemia yielded similar results, supporting the robustness of the findings (Supplementary Tables S6–S20). The E-value analysis indicated the magnitude of unmeasured confounding that would be required to explain away the observed associations; however, these estimates should not be interpreted as evidence of causality (Table 4).

**Table 3:** Associations between metabolic indices and SRCs in various subgroups.

Subgroup	*n*	OR (95% CI)	*P*	*P* for interaction	OR (95% CI)	*P*	*P* for interaction	OR (95% CI)	*P*	*P* for interaction
		**TG/HDL-C**			**TyG**			**TyG-WC**		
Sex				0.344			0.088			0.112
Female	14,416	1.26 (1.17–1.36)	<0.001		1.18 (1.11 –1.27)	<0.001		1.28 (1.19–1.37)	<0.001	
Male	20,584	1.18 (1.15–1.22)	<0.001		1.22 (1.17–1.27)	<0.001		1.39 (1.33–1.46)	<0.001	
Age (years)				0.29			0.062			0.087
<50	15,825	1.20 (1.15–1.24)	<0.001		1.23 (1.16–1.30)	<0.001		1.37 (1.28–1.46)	<0.001	
≥50	19,175	1.18 (1.13–1.23)	<0.001		1.17 (1.12–1.22)	<0.001		1.29 (1.23–1.35)	<0.001	
BMI (kg/m^2^)				0.125			0.094			0.089
<24	7,245	1.13 (1.02–1.24)	0.016		1.19 (1.08–1.31)	<0.001		1.15 (1.04–1.28)	0.005	
≥24	27,755	1.19 (1.16–1.22)	<0.001		1.20 (1.16–1.24)	<0.001		1.37 (1.32–1.43)	<0.001	
Current smoking				0.363			0.074			0.075
No	23,205	1.19 (1.14–1.24)	<0.001		1.18 (1.13–1.24)	<0.001		1.39 (1.33–1.46)	<0.001	
Yes	11,795	1.17 (1.13–1.22)	<0.001		1.23 (1.16–1.29)	<0.001		1.26 (1.18–1.34)	<0.001	
Current drinking				0.122			0.438			0.216
No	24,051	1.18 (1.14–1.23)	<0.001		1.22 (1.17–1.27)	<0.001		1.36 (1.30–1.42)	<0.001	
Yes	10,949	1.21 (1.17–1.26)	<0.001		1.22 (1.15–1.29)	<0.001		1.34 (1.25–1.44)	<0.001	
		**TyG-BMI**			**METS-IR**			**METS-VF**		
Sex				<0.001			0.117			0.140
Female	14,416	1.66 (1.57–1.75)	<0.001		1.50 (1.40–1.61)	<0.001		1.59 (1.47–1.72)	<0.001	
Male	20,584	1.30 (1.26–1.35)	<0.001		1.41 (1.36–1.47)	<0.001		1.51 (1.45–1.57)	<0.001	
Age (years)				0.264			0.319			0.934
<50	15,825	1.36 (1.29–1.43)	<0.001		1.39 (1.32–1.47)	<0.001		1.36 (1.28–1.45)	<0.001	
≥50	19,175	1.45 (1.40–1.51)	<0.001		1.51 (1.45–1.58)	<0.001		1.36 (1.30–1.42)	<0.001	
BMI (kg/m^2^)				0.105			0.779			0.056
<24	7,245	1.36 (1.30–1.43)	<0.001		1.84 (1.43–2.37)	<0.001		1.53 (1.35–1.73)	<0.001	
≥24	27,755	1.47 (1.41–1.52)	<0.001		1.31 (1.26–1.36)	<0.001		1.60 (1.54–1.67)	<0.001	
Current smoking				0.19			0.154			0.463
No	23,205	1.40 (1.35–1.45)	<0.001		1.43 (1.37–1.49)	<0.001		1.53 (1.46–1.60)	<0.001	
Yes	11,795	1.41 (1.34–1.49 )	<0.001		1.46 (1.38–1.55)	<0.001		1.53 (1.45–1.63)	<0.001	
Current drinking				0.838			0.992			0.001
No	24,051	1.39 (1.35–1.44)	<0.001		1.41 (1.35–1.46)	<0.001		1.45 (1.39–1.51)	<0.001	
Yes	10,949	1.43 (1.35–1.51)	<0.001		1.52 (1.43–1.62)	<0.001		1.77 (1.64–1.90)	<0.001	

ORs are presented per 1-SD increase in each metabolic index. Subgroup analyses were adjusted for all covariates included in Model 4, except for the corresponding stratification variable.

**Table 4:** E-value analysis for the associations between metabolic indices and SRCs.

Exposure	SRCs	Multiple SRCs	Large SRCs
	E-value	E-value	E-value
TG/HDL-C (Per SD increase)	1.67	1.34	1.69
TyG (Per SD increase)	1.76	1.43	1.86
TyG-WC (Per SD increase)	2.04	1.59	2.06
TyG-BMI (Per SD increase)	2.28	1.90	2.43
METS-IR (Per SD increase)	2.43	2.54	2.60
METS-VF (Per SD increase)	2.69	2.73	2.98

E-values were calculated using the fully adjusted OR point estimates from Model 4. Adjusted ORs are shown in Table 2 and Supplementary Tables S2, S3.

## Discussion

In this cross-sectional study, all six metabolic indices were positively associated with prevalent SRCs after multivariable adjustment, with consistent dose-response patterns in RCS analyses. Similar findings were observed for large and multiple SRCs. Among the indices evaluated, METS-VF showed the largest observed AUC. The associations were generally consistent across subgroup and sensitivity analyses. E-values ranged from 1.34 to 2.98, indicating the magnitude of unmeasured confounding required to explain away the observed associations. Notably, indices incorporating adiposity measures showed stronger associations with SRCs, possibly because they more comprehensively reflect the metabolic disturbances associated with adiposity and insulin resistance.

Although SRCs are often detected incidentally, accumulating evidence suggests that they may be related to metabolic abnormalities. Shen et al. reported a positive association between SRCs and metabolic syndrome in a large adult population, with stronger associations observed among individuals with larger or multiple cysts, suggesting that metabolic abnormalities may also be associated with greater cyst burden (7). In a cross-sectional study, reported that metabolic syndrome was associated with larger renal cyst size (11). This finding is consistent with our results, as all six metabolic indices were positively associated with large SRCs. Additional evidence from patients with type 2 diabetes supports the clinical relevance of SRCs in the setting of impaired glucose metabolism. Wei et al. reported poorer renal function among diabetic patients with SRCs, whereas Han et al. found that hyperuricemia and excessive urinary uric acid excretion were linked to a higher likelihood of SRCs in this population (12, 13). These findings suggest that routinely available metabolic indices may provide supplementary information for identifying hypertensive patients with higher odds of prevalent SRCs or greater cyst burden.

Several biological pathways may partly explain the observed association between metabolic dysfunction and prevalent SRCs. First, insulin resistance may be an important link. Metabolic indices such as TG/HDL-C, TyG, and related composite indices have been widely used as surrogate markers of metabolic disturbance (15, 17, 18). Insulin resistance may promote hyperinsulinemia, sympathetic activation, and renin—angiotensin—aldosterone system dysregulation, thereby increasing renal hemodynamic stress and contributing to arteriolar sclerosis and microcirculatory impairment (21–24). Chronic microvascular damage and local ischemia may disturb renal tubular epithelial homeostasis and promote tubular dilation, interstitial remodeling, and local fluid retention, thereby providing a plausible substrate for SRC formation (25, 26). Second, disturbances in lipid and glucose metabolism may also be involved. The indices evaluated in this study collectively reflect dyslipidemia, impaired glucose metabolism, insulin resistance, and adiposity-related metabolic burden. These abnormalities may promote oxidative stress, chronic low-grade inflammation, mitochondrial dysfunction, endothelial injury, and renal tubular epithelial damage, thereby providing a plausible biological context for the presence and greater burden of SRCs (17, 18, 27, 28). Such changes may create a renal microenvironment that favors tubular remodeling and fluid retention, providing a possible biological context for the presence of SRCs and greater cyst burden (28–31). In addition, perirenal and renal sinus fat accumulation may also influence renal structure through mechanical compression, local inflammatory responses, microcirculatory disturbance, and altered renal hemodynamics (32–35). These pathways may provide a plausible biological context for the presence of SRCs or greater cyst burden.

Several aspects of this work support the reliability of the analysis. The large sample size improved the precision of the estimates, and evaluating large and multiple SRCs separately allowed cyst burden to be characterized in greater detail. The metabolic indices used in this analysis were calculated from routinely collected laboratory and anthropometric data, which may facilitate their application in clinical and epidemiological research. In addition, the associations remained generally consistent across multivariable regression, RCS modeling, subgroup analyses, and sensitivity checks, supporting the internal coherence of the results.

However, this study still has several limitations. First, the cross-sectional design did not permit assessment of temporal sequence or causal relationships. Serial renal imaging and longitudinal metabolic data were unavailable; therefore, we could not evaluate changes in cyst size or number over time or determine whether cyst progression was associated with subsequent adverse metabolic outcomes. Second, all participants were hospitalized patients with hypertension, which may limit the generalizability of the findings to community-based populations or individuals without hypertension. Third, despite adjustment for multiple covariates, residual confounding from unmeasured or incompletely captured factors, such as dietary patterns, physical activity, and genetic susceptibility, cannot be excluded. Moreover, gamma-glutamyl transferase (GGT), an indicator of hepatobiliary and metabolic dysfunction, was not included in the present analysis; therefore, its potential confounding or modifying effect on the associations between metabolic indices and SRCs could not be evaluated. Fourth, although overall glucose-lowering medication use and major antihypertensive drug classes, including ACEIs/ARBs, were included as covariates, detailed information on specific agents, treatment indications, treatment duration, cumulative exposure, and adherence was not consistently available. In addition, serial renal imaging was unavailable. Therefore, the specific effects of agents such as SGLT-2 inhibitors or RAAS inhibitors on SRC formation or progression could not be evaluated. Finally, although the E-value analysis provided a quantitative assessment of potential unmeasured confounding, it did not establish causality or address reverse causation and temporal ambiguity arising from the cross-sectional design.

## Conclusion

TG/HDL-C, TyG, TyG-WC, TyG-BMI, METS-IR, and METS-VF were positively associated with prevalent SRCs after multivariable adjustment, with significant trends and generally consistent dose–response relationships. Similar associations were observed for large and multiple SRCs, while METS-VF showed the strongest association and the highest observed discriminative performance. These findings suggest that metabolic dysfunction may be relevant to SRC burden among patients with hypertension. Prospective studies are needed to confirm the temporal relationships and clinical relevance of these findings.

## Data Availability

The original contributions presented in the study are included in the article/Supplementary Material, further inquiries can be directed to the corresponding author.
