# Supplementary material for: Associations between metabolic indices and the prevalence of simple renal cysts among hospitalized patients with hypertension in Xinjiang, China: a large-scale cross-sectional study
Source: Front Cardiovasc Med. 2026 Aug 12;13:1911446. doi: 10.3389/fcvm.2026.1911446 (PMC13506526; doi:10.3389/fcvm.2026.1911446)
Supplement: Supplementary file 1 [file Datasheet1.doc]

**Supplementary Material**

**1. Supplemental material and methods**

**Anthropometric data**

Anthropometric data, including weight and height, were collected by trained nurses using a standardized protocol. Body weight was measured to the nearest 0.1 kg, while height was measured to the nearest 0.1 cm in bare feet. Body mass index (BMI) was calculated according to the formula: BMI = Weight (kg) / Height (m)². Waist circumference (WC) was measured using a standardized protocol: participants stood upright with feet approximately 25-30 cm apart, body weight evenly distributed, abdomen relaxed, and arms hanging naturally at the sides. A non-elastic measuring tape was placed horizontally around the abdomen at the midpoint between the lower margin of the last rib and the upper border of the iliac crest along the midaxillary line, ensuring the tape was snug but not compressing the skin. Measurements were taken at the end of normal expiration and recorded to the nearest 0.1 cm. Typically, two measurements were obtained and averaged; if a substantial discrepancy existed, a third measurement was performed, and the average of the two closest values was used. Current smokers were defined as individuals who had smoked at least 100 cigarettes in their lifetime and were currently smoking. Alcohol consumption was assessed according to self-reported current drinking status, including the type of alcoholic beverage, drinking frequency, and usual amount consumed per occasion. For the present analysis, participants were classified as current drinkers or non-current drinkers according to whether they reported current alcohol consumption at baseline. Blood pressure was measured using a mercury sphygmomanometer after the patient had rested quietly for at least 10 minutes. The average of the measurements was recorded as the SBP and DBP values.

**Biochemical parameters**

Biochemical parameters including measurements of alanine aminotransferase (ALT), aspartate aminotransferase (AST), creatinine (Cr), blood urea nitrogen (BUN), uric acid (UA), potassium, total cholesterol (TC), triglycerides (TG), high-density lipoprotein (HDL-C), low-density lipoprotein (LDL-C), fasting plasma glucose (FPG) and glycosylated hemoglobin (HbA1c) were measured using a C16000 automated biochemistry analyzer (Abbott Laboratories, Abbott Park, IL, USA).

**Definition of the disease**

Criteria for hypertension included self-reported hypertension, current use of anti-hypertensive medication, or SBP ≥ 140 mmHg and/or DBP ≥ 90 mmHg recorded for at least three consecutive readings. CHD was defined as a fatal or nonfatal myocardial infarction, unstable angina, and coronary revascularization. Diabetes mellitus was defined by several criteria, including the past and current use of any hypoglycemic drug, a fasting blood glucose level greater than 7.0 mmol/L, a 2-hour oral glucose tolerance test blood glucose level greater than or equal to 11.1 mmol/L, or a random blood glucose level greater than or equal to 11.1 mmol/L. Diabetes mellitus was diagnosed if any of these criteria were met. According to National Cholesterol Education Program (Adult Treatment Panel III), the definition of dyslipidemia was shown below: hypercholesterolemia (TC ≥ 200 mg/dL), hypertriglyceridemia (TG ≥ 150 mg/dL), low HDL-cholesterolemia (HDL-C ≤ 40 mg/dL in men, and ≤ 50 mg/dL in women), and high LDL-cholesterolemia (LDL-C ≥ 130 mg/dL). Hyperuricemia was defined as a serum uric acid level >420 μmol/L in men or >360 μmol/L in women.

**Diagnosis of SRC**

SRCs were assessed by certified radiologists using color Doppler ultrasonography (IU22; Philips Healthcare, Andover, MA, USA) or abdominal CT (SOMATOM Definition Flash; Siemens Healthineers, Germany). On ultrasonography, SRCs were identified as round or oval, homogeneous anechoic lesions with a thin smooth wall, clear margins, and posterior acoustic enhancement. On CT, they were characterized by homogeneous water-like attenuation (−5 to +15 Hounsfield units), a thin imperceptible wall, well-defined margins, and no post-contrast enhancement.

**Statistical analysis**

Missing covariate data were handled using multiple imputation by chained equations with the “mice” package in R, generating five imputed datasets. Baseline characteristics were presented as n (%) if categorical, mean (SD) if normally distributed, and median (interquartile range) if nonnormally distributed. Baseline characteristics were compared using the chi-square test for categorical variables, the independent-samples Student’s t-test for normally distributed continuous variables, and the Mann–Whitney U test for nonnormally distributed continuous variables. Prior to any regression analysis, we tested multicollinearity among the predictors by checking for the variance inflation factor. In the initial collinearity assessment, TC and LDL-C showed high VIF values and were therefore not retained in the final multivariable model. After their exclusion, all retained covariates had VIF values below 10, suggesting that the final regression models were not materially affected by multicollinearity. The associations between metabolic indices and SRCs were examined using multivariable logistic regression models. Four models were constructed, and each metabolic index was analyzed both as a continuous variable and as a categorical variable based on quartiles. Restricted cubic spline models with four knots at the 5th, 35th, 65th, and 95th percentiles were used to evaluate dose-response relationships between metabolic indices and the odds of prevalent SRCs. In addition, subgroup analysis was conducted by the stratified multiple regression method according to sex (female or male), age (< 50 or ≥ 50 years), BMI (< 24 or ≥ 24kg/m^2^), current smoking (Yes or No), current drinking (Yes or No). We performed a series of sensitivity analyses by separately excluding participants with DM, BMI ≥30 kg/m², nephrolithiasis, hyperuricemia, or dyslipidemia. All analyses were performed using R version 4.5.1. All p-values were two-sided, and p-values of less than 0.05 were considered statistically significant.

2 Supplementary Figures and Tables

## 2.1 Supplementary Tables

Table S1. Collinearity diagnostics of the final multivariable models.

| Variable | Step1 | Step2 |
| --- | --- | --- |
| sex | 1.843523 | 1.841476 |
| age | 1.831505 | 1.821306 |
| BMI | 1.121988 | 1.116178 |
| SBP | 1.943007 | 1.938449 |
| DBP | 2.36183 | 2.36089 |
| Current smoking | 1.669939 | 1.66925 |
| Current drinking | 1.573841 | 1.57352 |
| SCr | 1.156509 | 1.153918 |
| ALT | 5.741781 | 5.730961 |
| AST | 5.415258 | 5.415186 |
| UA | 2.500614 | 2.482049 |
| TC | 13.309486 | / |
| TG | 3.941968 | 1.2648 |
| HDL-C | 2.122992 | 1.255929 |
| LDL-C | 10.294546 | / |
| FPG | 1.646932 | 1.643573 |
| eGFR | 1.133115 | 1.133022 |
| DM | 1.643087 | 1.639949 |
| Dyslipidemia | 1.126092 | 1.070856 |
| CHD | 1.240209 | 1.24008 |
| Nephrolith | 1.030271 | 1.030166 |
| Hyperuricemia | 1.925611 | 1.9252 |
| ACEI/ARBs | 1.262498 | 1.260629 |
| Beta blocker | 1.129893 | 1.129342 |
| CCB | 1.138004 | 1.136264 |
| Diuretics | 1.177602 | 1.176715 |
| Lipid.lowering.drugs | 1.794019 | 1.748382 |
| Antidiabetic | 1.826115 | 1.825807 |

Abbreviations: WC, waist circumference; BMI, body mass index; SBP, systolic blood pressure; DBP, diastolic blood

pressure; ALT, alanine transaminase; AST, aspartate transaminase; SCr, serum creatinine; eGFR, estimated glomerular filtration rate; TC, total cholesterol; TG, triglyceride; HDL-C, high-density lipoprotein cholesterol; LDL-C, low-density lipoprotein cholesterol; FPG, fasting plasma glucose; UA, uric acid; TyG, triglyceride-glucose; METS-IR, metabolic score for insulin resistance; METS-VF, metabolic score for visceral fat; DM, diabetes mellitus; CHD, coronary heart disease; ARBs, angiotensin receptor blockers; ACEIs, angiotensin-converting

enzyme inhibitors.

Table S2 Associations between metabolic indices and large SRCs.

| Variables | Model 1 | Model 2 | Model 3 | Model 4 |
| --- | --- | --- | --- | --- |
|  | OR (95% CI) | OR (95% CI) | OR (95% CI) | OR (95% CI) |
| **TG/HDL-C** |  |  |  |  |
| Per SD increase | 1.26 (1.22 - 1.31) | 1.20 (1.15 - 1.25) | 1.20 (1.15 - 1.25) | 1.20 (1.15 - 1.25) |
| Quartiles |  |  |  |  |
| Q1 | Reference | Reference | Reference | Reference |
| Q2 | 1.48 (1.26 - 1.73) | 1.20 (1.02 - 1.41) | 1.20 (1.02 - 1.41) | 1.21 (1.03 - 1.42) |
| Q3 | 1.59 (1.36 - 1.86) | 1.15 (0.98 - 1.36) | 1.15 (0.98 - 1.36) | 1.16 (0.98 - 1.36) |
| Q4 | 2.54 (2.19 - 2.94) | 1.73 (1.47 - 2.03) | 1.73 (1.47 - 2.03) | 1.73 (1.47 - 2.04) |
| P for trend | <0.001 | <0.001 | 0.001 | <0.001 |
| TyG |  |  |  |  |
| Per SD increase | 1.46 (1.40 - 1.53) | 1.29 (1.23 - 1.36) | 1.29 (1.23 - 1.36) | 1.27 (1.20 - 1.35) |
| Quartiles |  |  |  |  |
| Q1 | Reference | Reference | Reference | Reference |
| Q2 | 1.66 (1.41 - 1.95) | 1.25 (1.06 - 1.48) | 1.25 (1.06 - 1.48) | 1.25 (1.05 - 1.48) |
| Q3 | 1.78 (1.51 - 2.09) | 1.34 (1.14 - 1.59) | 1.34 (1.14 - 1.59) | 1.34 (1.13 - 1.58) |
| Q4 | 2.91 (2.51 - 3.38) | 1.75 (1.49 - 2.08) | 1.75 (1.49 - 2.08) | 1.75 (1.47 - 2.07) |
| P for trend | <0.001 | <0.001 | <0.001 | <0.001 |
| **TyG-WC** |  |  |  |  |
| Per SD increase | 1.54 (1.45 - 1.64) | 1.38 (1.30 - 1.47) | 1.38 (1.30 - 1.47) | 1.36 (1.27 - 1.45) |
| Quartiles |  |  |  |  |
| Q1 | Reference | Reference | Reference | Reference |
| Q2 | 2.62 (2.22 - 3.11) | 2.33 (1.96 - 2.76) | 2.33 (1.96 - 2.76) | 2.30 (1.94 - 2.74) |
| Q3 | 3.95 (3.37 - 4.65) | 3.30 (2.80 - 3.89) | 3.30 (2.80 - 3.89) | 3.22 (2.73 - 3.81) |
| Q4 | 3.58 (3.06 - 4.20) | 3.18 (2.72 - 3.74) | 2.95 (2.51 - 3.47) | 2.97 (2.52 - 3.50) |
| P for trend | <0.001 | <0.001 | <0.001 | <0.001 |
| **TyG-BMI** |  |  |  |  |
| Per SD increase | 1.62 (1.55 - 1.69) | 1.54 (1.47 - 1.62) | 1.54 (1.47 - 1.62) | 1.53 (1.46 - 1.61) |
| Quartiles |  |  |  |  |
| Q1 | Reference | Reference | Reference | Reference |
| Q2 | 1.61 (1.33 - 1.97) | 1.35 (1.10 - 1.65) | 1.35 (1.10 - 1.65) | 1.34 (1.09 - 1.64) |
| Q3 | 3.43 (2.88 - 4.11) | 2.86 (2.38 - 3.45) | 2.86 (2.38 - 3.45) | 2.83 (2.35 - 3.42) |
| Q4 | 5.06 (4.28 - 6.03) | 4.32 (3.60 - 5.21) | 4.32 (3.60 - 5.21) | 4.24 (3.53 - 5.12) |
| P for trend | <0.001 | <0.001 | <0.001 | <0.001 |
| **METS-IR** |  |  |  |  |
| Per SD increase | 1.64 (1.57 - 1.72) | 1.62 (1.55 - 1.70) | 1.62 (1.55 - 1.70) | 1.61 (1.53 - 1.69) |
| Quartiles |  |  |  |  |
| Q1 | Reference | Reference | Reference | Reference |
| Q2 | 1.42 (1.15 - 1.76) | 1.31 (1.05 - 1.63) | 1.31 (1.05 - 1.63) | 1.30 (1.04 - 1.62) |
| Q3 | 3.93 (3.28 - 4.73) | 3.82 (3.13 - 4.68) | 3.82 (3.13 - 4.68) | 3.79 (3.10 - 4.65) |
| Q4 | 5.79 (4.86 - 6.93) | 5.94 (4.84 - 7.33) | 5.94 (4.84 - 7.33) | 5.84 (4.74 - 7.22) |
| P for trend | <0.001 | <0.001 | <0.001 | <0.001 |
| **METS-VF** |  |  |  |  |
| Per SD increase | 1.84 (1.72 - 1.97) | 1.82 (1.69 - 1.97) | 1.82 (1.69 - 1.97) | 1.79 (1.66 - 1.94) |
| Quartiles |  |  |  |  |
| Q1 | Reference | Reference | Reference | Reference |
| Q2 | 1.51 (1.22 - 1.87) | 1.56 (1.26 - 1.93) | 1.56 (1.26 - 1.93) | 1.56 (1.26 - 1.93) |
| Q3 | 3.74 (3.11 - 4.52) | 3.89 (3.23 - 4.71) | 3.89 (3.23 - 4.71) | 3.86 (3.21 - 4.68) |
| Q4 | 6.44 (5.40 - 7.73) | 6.47 (5.40 - 7.82) | 6.47 (5.40 - 7.82) | 6.38 (5.31 - 7.71) |
| P for trend | <0.001 | <0.001 | <0.001 | <0.001 |

Model 1 was unadjusted;

Model 2 was adjusted for age, sex, BMI, current smoking, current drinking, SBP, DBP;

Model 3 was further adjusted for ALT, AST, TG, HDL-C, eGFR, UA, FPG;

Model 4 was additionally adjusted for DM, dyslipidemia, CHD, nephrolithiasis, hyperuricemia, lipid-lowering drugs, antidiabetic agents, diuretics, beta-blockers, calcium channel blockers, and ACEI/ARBs.

Abbreviations: WC, waist circumference; BMI, body mass index; TyG, triglyceride-glucose; METS-IR, metabolic score for insulin resistance; METS-VF, metabolic score for visceral fat; OR, odds ratio; CI, confidence interval; Other abbreviations, see Table 1.

Table S3 Associations between metabolic indices and multiple SRCs.

| Variables | Model 1 | Model 2 | Model 3 | Model 4 |
| --- | --- | --- | --- | --- |
|  | OR (95% CI) | OR (95% CI) | OR (95% CI) | OR (95% CI) |
| **TG/HDL-C** |  |  |  |  |
| Per SD increase | 1.14 (1.10 - 1.18) | 1.10 (1.05 - 1.15) | 1.08 (1.02 - 1.13) | 1.07 (1.02 - 1.12) |
| Quartiles |  |  |  |  |
| Q1 | Reference | Reference | Reference | Reference |
| Q2 | 1.36 (1.17 - 1.59) | 1.15 (0.98 - 1.34) | 1.13 (0.97 - 1.32) | 1.13 (0.96 - 1.32) |
| Q3 | 1.71 (1.47 - 1.98) | 1.34 (1.15 - 1.56) | 1.30 (1.11 - 1.52) | 1.28 (1.10 - 1.50) |
| Q4 | 1.88 (1.63 - 2.18) | 1.45 (1.25 - 1.70) | 1.37 (1.17 - 1.61) | 1.35 (1.15 - 1.59) |
| P for trend | <0.001 | <0.001 | <0.001 | <0.001 |
| **TyG** |  |  |  |  |
| Per SD increase | 1.27 (1.22 - 1.33) | 1.16 (1.10 - 1.22) | 1.11 (1.06 - 1.17) | 1.10 (1.04 - 1.17) |
| Quartiles |  |  |  |  |
| Q1 | Reference | Reference | Reference | Reference |
| Q2 | 1.61 (1.38 - 1.89) | 1.41 (1.20 - 1.65) | 1.34 (1.14 - 1.57) | 1.33 (1.13 - 1.56) |
| Q3 | 1.85 (1.59 - 2.16) | 1.49 (1.27 - 1.74) | 1.38 (1.18 - 1.62) | 1.36 (1.15 - 1.59) |
| Q4 | 2.19 (1.89 - 2.54) | 1.62 (1.39 - 1.89) | 1.45 (1.24 - 1.71) | 1.41 (1.19 - 1.67) |
| P for trend | <0.001 | <0.001 | <0.001 | <0.001 |
| **TyG-WC** |  |  |  |  |
| Per SD increase | 1.30 (1.22 - 1.38) | 1.21 (1.14 - 1.28) | 1.18 (1.12 - 1.25) | 1.16 (1.11 - 1.22) |
| Quartiles |  |  |  |  |
| Q1 | Reference | Reference | Reference | Reference |
| Q2 | 1.67 (1.40 - 1.99) | 1.61 (1.35 - 1.92) | 1.59 (1.33 - 1.90) | 1.58 (1.33 - 1.89) |
| Q3 | 2.57 (2.18 - 3.03) | 2.37 (2.01 - 2.80) | 2.32 (1.97 - 2.75) | 2.30 (1.95 - 2.72) |
| Q4 | 3.44 (2.94 - 4.04) | 3.08 (2.63 - 3.62) | 2.95 (2.51 - 3.47) | 2.86 (2.43 - 3.37) |
| P for trend | <0.001 | <0.001 | <0.001 | <0.001 |
| **TyG-BMI** |  |  |  |  |
| Per SD increase | 1.53 (1.46 - 1.60) | 1.44 (1.27 - 1.64) | 1.32 (1.15 - 1.50) | 1.29 (1.12 - 1.50) |
| Quartiles |  |  |  |  |
| Q1 | Reference | Reference | Reference | Reference |
| Q2 | 1.48 (1.22 - 1.80) | 1.34 (1.11 - 1.63) | 1.28 (1.05 - 1.56) | 1.26 (1.04 - 1.54) |
| Q3 | 3.60 (3.05 - 4.27) | 3.41 (2.88 - 4.07) | 3.20 (2.68 - 3.82) | 3.14 (2.64 - 3.76) |
| Q4 | 4.02 (3.41 - 4.77) | 4.02 (3.39 - 4.79) | 3.71 (3.11 - 4.45) | 3.60 (3.01 - 4.33) |
| P for trend | <0.001 | <0.001 | <0.001 | <0.001 |
| **METS-IR** |  |  |  |  |
| Per SD increase | 1.57 (1.51 - 1.65) | 1.59 (1.52 - 1.67) | 1.59 (1.52 - 1.67) | 1.58 (1.50 - 1.66) |
| Quartiles |  |  |  |  |
| Q1 | Reference | Reference | Reference | Reference |
| Q2 | 1.45 (1.19 - 1.78) | 1.32 (1.07 - 1.62) | 1.37 (1.11 - 1.69) | 1.36 (1.10 - 1.68) |
| Q3 | 3.57 (3.00 - 4.27) | 3.42 (2.86 - 4.10) | 3.64 (3.00 - 4.44) | 3.59 (2.95 - 4.38) |
| Q4 | 5.15 (4.35 - 6.10) | 5.19 (4.35 - 6.21) | 5.63 (4.62 - 6.91) | 5.51 (4.50 - 6.78) |
| P for trend | <0.001 | <0.001 | <0.001 | <0.001 |
| **METS-VF** |  |  |  |  |
| Per SD increase | 1.72 (1.61 - 1.84) | 1.70 (1.58 - 1.84) | 1.71 (1.59 - 1.85) | 1.67 (1.55 - 1.80) |
| Quartiles |  |  |  |  |
| Q1 | Reference | Reference | Reference | Reference |
| Q2 | 2.01 (1.64 - 2.46) | 1.97 (1.62 - 2.42) | 2.05 (1.67 - 2.51) | 2.05 (1.68 - 2.52) |
| Q3 | 3.62 (3.01 - 4.38) | 3.51 (2.92 - 4.25) | 3.68 (3.06 - 4.46) | 3.64 (3.03 - 4.42) |
| Q4 | 6.03 (5.06 - 7.24) | 5.64 (4.71 - 6.79) | 5.87 (4.89 - 7.08) | 5.68 (4.73 - 6.92) |
| P for trend | <0.001 | <0.001 | <0.001 | <0.001 |

Model 1 was unadjusted;

Model 2 was adjusted for age, sex, BMI, current smoking, current drinking, SBP, DBP;

Model 3 was further adjusted for ALT, AST, TG, HDL-C, eGFR, UA, FPG;

Model 4 was additionally adjusted for DM, dyslipidemia, CHD, nephrolithiasis, hyperuricemia, lipid-lowering drugs, antidiabetic agents, diuretics, beta-blockers, calcium channel blockers, and ACEI/ARBs.

Abbreviations: WC, waist circumference; BMI, body mass index; TyG, triglyceride-glucose; METS-IR, metabolic score for insulin resistance; METS-VF, metabolic score for visceral fat; OR, odds ratio; CI, confidence interval; Other abbreviations, see Table 1.

Table S4 Association between metabolic indices and large SRCs in various subgroups.

| Subgroup | n | OR (95% CI) | *P* | P for interaction | OR (95% CI) | *P* | P for interaction | OR (95% CI) | *P* | P for interaction |
| --- | --- | --- | --- | --- | --- | --- | --- | --- | --- | --- |
|  |  |  |  |  |  |  |  |  |  |  |
| Sex |  |  |  | 0.318 |  |  | 0.180 |  |  | 0.094 |
| Female | 14416 | 1.11 (0.97 - 1.27) | 0.146 |  | 1.28 (1.11 - 1.48) | 0.001 |  | 1.21 (1.05 - 1.40) | 0.01 |  |
| Male | 20584 | 1.19 (1.14 -1.24) | <0.001 |  | 1.39 (1.29 - 1.50) | <0.001 |  | 1.41 (1.31 - 1.52) | <0.001 |  |
| Age (years) |  |  |  | 0.142 |  |  | 0.229 |  |  | 0.576 |
| <50 | 15825 | 1.13 (1.06 -1.20) | <0.001 |  | 1.31 (1.19 - 1.45) | <0.001 |  | 1.42 (1.27 - 1.59) | <0.001 |  |
| ≥50 | 19175 | 1.20 (1.14 -1.27) | <0.001 |  | 1.27 (1.17 - 1.37) | <0.001 |  | 1.32 (1.22 - 1.43) | <0.001 |  |
| BMI (kg/m^2^) |  |  |  | 0.339 |  |  | 0.139 |  |  | 0.029 |
| <24 | 7245 | 1.12 (0.93 -1.34) | 0.23 |  | 1.26 (1.03 - 1.53) | 0.021 |  | 1.23 (1.13 - 1.34) | <0.001 |  |
| ≥24 | 27755 | 1.18 (1.14 -1.23) | <0.001 |  | 1.30 (1.22 - 1.39) | <0.001 |  | 1.37 (1.32 - 1.42) | <0.001 |  |
| Current smoking |  |  |  | 0.519 |  |  | 0.181 |  |  | 0.075 |
| No | 23205 | 1.16 (1.09 -1.24) | <0.001 |  | 1.24 (1.13 - 1.34) | <0.001 |  | 1.40 (1.32 - 1.48) | <0.001 |  |
| Yes | 11795 | 1.22 (1.16 - 1.28) | <0.001 |  | 1.41 (1.29 -1.54 ) | <0.001 |  | 1.31 (1.26 - 1.37) | <0.001 |  |
| Current drinking |  |  |  | 0.097 |  |  | 0.203 |  |  | 0.114 |
| No | 24051 | 1.13 (1.06 - 1.20) | 0.006 |  | 1.25 (1.16 - 1.36) | <0.001 |  | 1.31 (1.20 - 1.42) | <0.001 |  |
| Yes | 10949 | 1.25 (1.19 - 1.31) | <0.001 |  | 1.39 (1.26 - 1.53) | <0.001 |  | 1.47 (1.32 - 1.64) | <0.001 |  |
|  |  | **TyG-BMI** |  |  | **METS-IR** |  |  | **METS-VF** |  |  |
| Sex |  |  |  | 0.066 |  |  | 0.588 |  |  | 0.367 |
| Female | 14416 | 1.62 (1.45 - 1.80) | <0.001 |  | 1.60 (1.43 - 1.79) | <0.001 |  | 1.43 (1.25 - 1.63) | <0.001 |  |
| Male | 20584 | 1.53 (1.44 - 1.62) | <0.001 |  | 1.51 (1.42 - 1.59) | <0.001 |  | 1.31 (1.22 - 1.40) | <0.001 |  |
| Age (years) |  |  |  | 0.917 |  |  | 0.054 |  |  | 0.942 |
| <50 | 15825 | 1.51 (1.38 - 1.64) | <0.001 |  | 1.51 (1.39 - 1.64) | <0.001 |  | 1.29 (1.17 -1.42) | <0.001 |  |
| ≥50 | 19175 | 1.56 (1.47 - 1.66 ) | <0.001 |  | 1.59 (1.49 - 1.69) | <0.001 |  | 1.47 (1.35 - 1.60) | <0.001 |  |
| BMI (kg/m^2^) |  |  |  | 0.167 |  |  | 0.104 |  |  | 0.054 |
| <24 | 7245 | 1.63 (1.14 - 2.35) | 0.008 |  | 2.19 (1.59 - 3.01) | <0.001 |  | 1.20 (1.05 - 1.37) | 0.008 |  |
| ≥24 | 27755 | 1.52 (1.44 - 1.61) | <0.001 |  | 1.48 (1.39 - 1.56) | <0.001 |  | 1.53 (1.42 - 1.64) | <0.001 |  |
| Current smoking |  |  |  | 0.616 |  |  | 0.088 |  |  | 0.064 |
| No | 23205 | 1.73 (1.42 - 2.10) | <0.001 |  | 1.52 (1.42 - 1.63) | <0.001 |  | 1.54 (1.40 - 1.69) | <0.001 |  |
| Yes | 11795 | 1.98 (1.62 - 2.43) | <0.001 |  | 1.73 (1.60 - 1.87) | <0.001 |  | 1.23 (1.14 - 1.32) | <0.001 |  |
| Current drinking |  |  |  | 0.616 |  |  | 0.173 |  |  | 0.444 |
| No | 24051 | 1.72 (1.43 - 2.07) | <0.001 |  | 1.55 (1.45 - 1.65) | <0.001 |  | 1.38 (1.27 - 1.50) | <0.001 |  |
| Yes | 10949 | 1.98 (1.60 - 2.45) | <0.001 |  | 1.72 (1.58 - 1.87) | <0.001 |  | 1.39 (1.24 - 1.55) | <0.001 |  |

ORs are presented per 1-SD increase in each metabolic index. Subgroup analyses were adjusted for all covariates included in Model 4, except for the corresponding stratification variable.

Table S5 Association between metabolic indices and multiple SRCs in various subgroups.

| Subgroup | n | OR (95% CI) | *P* | P for interaction | OR (95% CI) | *P* | P for interaction | OR (95% CI) | *P* | P for interaction |
| --- | --- | --- | --- | --- | --- | --- | --- | --- | --- | --- |
|  |  | **TG/HDL-C** |  |  | **TyG** |  |  | **TyG-WC** |  |  |
| Sex |  |  |  | 0.102 |  |  | 0.065 |  |  | 0.136 |
| Female | 14416 | 0.96 (0.81 - 1.13) | 0.619 |  | 1.03 (0.91 -1.16) | 0.643 |  | 1.32 (1.15 - 1.51) | <0.001 |  |
| Male | 20584 | 1.07 (1.01 -1.12) | 0.012 |  | 1.13 (1.06 -1.21) | <0.001 |  | 1.15 (1.10 - 1.22) | <0.001 |  |
| Age (years) |  |  |  | 0.265 |  |  | 0.089 |  |  | 0.382 |
| <50 | 15825 | 1.07 (1.00 -1.14) | 0.044 |  | 1.15 (1.04 -1.27) | 0.006 |  | 1.17 (1.07 - 1.27) | 0.001 |  |
| ≥50 | 19175 | 1.00 (0.93 -1.07) | 0.932 |  | 1.10 (1.03 -1.18) | 0.006 |  | 1.16 (1.09 -1.24) | <0.001 |  |
| BMI (kg/m^2^) |  |  |  | 0.510 |  |  | 0.237 |  |  | 0.131 |
| <24 | 7245 | 1.17 (0.98 - 1.41) | 0.085 |  | 1.16 (0.97 -1.4) | 0.110 |  | 1.07 (1.01 - 1.14) | 0.032 |  |
| ≥24 | 27755 | 1.05 (1.00 -1.10) | 0.045 |  | 1.09 (1.03 -1.15) | 0.005 |  | 1.21 (1.13 - 1.29) | <0.001 |  |
| Current smoking |  |  |  | 0.752 |  |  | 0.085 |  |  | 0.131 |
| No | 23205 | 1.16 (1.09 -1.24) | <0.001 |  | 1.12 (1.04 -1.20) | 0.003 |  | 1.21 (1.11 - 1.31) | <0.001 |  |
| Yes | 11795 | 1.08 (1.02 - 1.15) | 0.007 |  | 1.11 (1.02 -1.21) | 0.015 |  | 1.16 (1.08 - 1.23) | <0.001 |  |
| Current drinking |  |  |  | 0.106 |  |  | 0.246 |  |  | 0.154 |
| No | 24051 | 1.10 (1.03 - 1.17) | 0.005 |  | 1.11 (1.03 -1.20) | 0.006 |  | 1.14 (1.08 -1.21) | <0.001 |  |
| Yes | 10949 | 1.06 (0.99 -1.13) | 0.083 |  | 1.13 (1.04 - 1.23) | 0.005 |  | 1.27 (1.14 - 1.41) | <0.001 |  |
|  |  | **TyG-BMI** |  |  | **METS-IR** |  |  | **METS-VF** |  |  |
| Sex |  |  |  | 0.987 |  |  | 0.585 |  |  | 0.257 |
| Female | 14416 | 1.46 (1.32 - 1.62) | <0.001 |  | 1.57 (1.39 - 1.77) | <0.001 |  | 1.45 (1.26 - 1.66) | <0.001 |  |
| Male | 20584 | 1.44 (1.36 - 1.52) | <0.001 |  | 1.49 (1.40 - 1.59) | <0.001 |  | 1.35 (1.22 - 1.48) | <0.001 |  |
| Age (years) |  |  |  | 0.091 |  |  | 0.015 |  |  | 0.063 |
| <50 | 15825 | 1.44 (1.32 - 1.56) | <0.001 |  | 1.48 (1.35 - 1.62) | <0.001 |  | 1.16 (1.03 - 1.31) | 0.017 |  |
| ≥50 | 19175 | 1.52 (1.43 - 1.62) | <0.001 |  | 1.62 (1.51 - 1.74) | <0.001 |  | 1.39 (1.26 - 1.53) | <0.001 |  |
| BMI (kg/m^2^) |  |  |  | 0.179 |  |  | 0.145 |  |  | 0.147 |
| <24 | 7245 | 2.23 (1.54 - 3.22) | <0.001 |  | 2.78 (1.86 - 4.16) | <0.001 |  | 1.23 (1.05 - 1.43) | 0.010 |  |
| ≥24 | 27755 | 1.36 (1.29 - 1.44) | <0.001 |  | 1.41 (1.32 - 1.50) | <0.001 |  | 1.46 (1.34 - 1.59) | <0.001 |  |
| Current smoking |  |  |  | 0.977 |  |  | 0.253 |  |  | <0.001 |
| No | 23205 | 1.45 (1.36 - 1.55) | <0.001 |  | 1.54 (1.43 - 1.65) | <0.001 |  | 1.45 (1.32 - 1.58) | <0.001 |  |
| Yes | 11795 | 1.53 (1.41 - 1.66) | <0.001 |  | 1.58 (1.44 - 1.73) | <0.001 |  | 1.50 (1.25 - 1.79) | <0.001 |  |
| Current drinking |  |  |  | 0.787 |  |  | 0.443 |  |  | 0.123 |
| No | 24051 | 1.46 (1.37 - 1.55) | <0.001 |  | 1.52 (1.42 - 1.63) | <0.001 |  | 1.29 (1.20 - 1.39) | <0.001 |  |
| Yes | 10949 | 1.53 (1.40 - 1.67) | <0.001 |  | 1.62 (1.47 - 1.79) | <0.001 |  | 1.95 (1.59 - 2.39) | <0.001 |  |

ORs are presented per 1-SD increase in each metabolic index. Subgroup analyses were adjusted for all covariates included in Model 4, except for the corresponding stratification variable.

Table S6. Sensitivity analysis of the associations between metabolic indices and SRCs was performed after excluding participants with BMI ≥ 30kg/m^2^.

| Variables | Model 1 | Model 2 | Model 3 | Model 4 |
| --- | --- | --- | --- | --- |
|  | OR (95% CI) | OR (95% CI) | OR (95% CI) | OR (95% CI) |
| **TG/HDL-C** |  |  |  |  |
| Per SD increase | 1.19 (1.16 - 1.22) | 1.15 (1.12 - 1.18) | 1.13 (1.10 - 1.17) | 1.12 (1.08 - 1.15) |
| Quartiles |  |  |  |  |
| Q1 | Reference | Reference | Reference | Reference |
| Q2 | 1.26 (1.16 - 1.37) | 1.10 (1.01 - 1.19) | 1.07 (0.98 - 1.16) | 1.06 (0.97 - 1.15) |
| Q3 | 1.42 (1.31 - 1.54) | 1.20 (1.10 - 1.30) | 1.13 (1.04 - 1.23) | 1.12 (1.02 - 1.22) |
| Q4 | 1.64 (1.51 - 1.78) | 1.37 (1.26 - 1.50) | 1.25 (1.14 - 1.37) | 1.23 (1.12 - 1.35) |
| P for trend | <0.001 | <0.001 | <0.001 | <0.001 |
| **TyG** |  |  |  |  |
| Per SD increase | 1.26 (1.22 - 1.29) | 1.21 (1.17 - 1.24) | 1.20 (1.16 - 1.23) | 1.15 (1.11 - 1.19) |
| Quartiles |  |  |  |  |
| Q1 | Reference | Reference | Reference | Reference |
| Q2 | 1.25 (1.15 - 1.35) | 1.20 (1.11 - 1.31) | 1.18 (1.09 - 1.29) | 1.15 (1.06 - 1.25) |
| Q3 | 1.44 (1.32 - 1.56) | 1.34 (1.23 - 1.46) | 1.31 (1.20 - 1.43) | 1.23 (1.13 - 1.35) |
| Q4 | 1.73 (1.60 - 1.88) | 1.55 (1.43 - 1.69) | 1.50 (1.37 - 1.63) | 1.33 (1.21 - 1.46) |
| P for trend | <0.001 | <0.001 | <0.001 | <0.001 |
| **TyG-WC** |  |  |  |  |
| Per SD increase | 1.38 (1.33 - 1.43) | 1.34 (1.29 - 1.39) | 1.33 (1.27 - 1.38) | 1.29 (1.24 - 1.34) |
| Quartiles |  |  |  |  |
| Q1 | Reference | Reference | Reference | Reference |
| Q2 | 1.34 (1.23 - 1.46) | 1.30 (1.20 - 1.42) | 1.30 (1.19 - 1.42) | 1.28 (1.18 - 1.40) |
| Q3 | 1.66 (1.52 - 1.80) | 1.61 (1.48 - 1.75) | 1.59 (1.46 - 1.73) | 1.55 (1.42 - 1.68) |
| Q4 | 1.82 (1.67 - 1.97) | 1.71 (1.57 - 1.86) | 1.69 (1.55 - 1.83) | 1.60 (1.47 - 1.74) |
| P for trend | <0.001 | <0.001 | <0.001 | <0.001 |
| **TyG-BMI** |  |  |  |  |
| Per SD increase | 1.58 (1.51 - 1.65) | 1.45 (1.33 - 1.59) | 1.45 (1.33 - 1.59) | 1.38 (1.25 - 1.52) |
| Quartiles |  |  |  |  |
| Q1 | Reference | Reference | Reference | Reference |
| Q2 | 1.41 (1.31 - 1.52) | 1.35 (1.25 - 1.45) | 1.34 (1.24 - 1.44) | 1.29 (1.19 - 1.39) |
| Q3 | 1.48 (1.38 - 1.60) | 1.38 (1.27 - 1.49) | 1.37 (1.26 - 1.49) | 1.28 (1.18 - 1.40) |
| Q4 | 2.86 (2.58 - 3.18) | 2.58 (2.32 - 2.88) | 2.56 (2.29 - 2.87) | 2.29 (2.04 - 2.57) |
| P for trend | <0.001 | <0.001 | <0.001 | <0.001 |
| **METS-IR** |  |  |  |  |
| Per SD increase | 1.58 (1.52 - 1.65) | 1.47 (1.41 - 1.54) | 1.47 (1.41 - 1.54) | 1.45 (1.38 - 1.52) |
| Quartiles |  |  |  |  |
| Q1 | Reference | Reference | Reference | Reference |
| Q2 | 1.32 (1.22 - 1.42) | 1.27 (1.17 - 1.37) | 1.27 (1.18 - 1.38) | 1.22 (1.13 - 1.32) |
| Q3 | 1.87 (1.73 - 2.02) | 1.76 (1.62 - 1.90) | 1.78 (1.64 - 1.93) | 1.67 (1.54 - 1.82) |
| Q4 | 2.91 (2.64 - 3.21) | 2.65 (2.39 - 2.94) | 2.71 (2.43 - 3.02) | 2.46 (2.21 - 2.75) |
| P for trend | <0.001 | <0.001 | <0.001 | <0.001 |
| **METS-VF** |  |  |  |  |
| Per SD increase | 1.86 (1.78 - 1.93) | 1.58 (1.50 - 1.66) | 1.56 (1.48 - 1.63) | 1.55 (1.47 - 1.63) |
| Quartiles |  |  |  |  |
| Q1 | Reference | Reference | Reference | Reference |
| Q2 | 1.77 (1.60 - 1.95) | 1.78 (1.61 - 1.98) | 1.80 (1.62 - 1.99) | 1.79 (1.61 - 1.98) |
| Q3 | 2.50 (2.27 - 2.75) | 2.48 (2.25 - 2.73) | 2.46 (2.23 - 2.71) | 2.42 (2.19 - 2.67) |
| Q4 | 3.92 (3.57 - 4.31) | 3.66 (3.32 - 4.03) | 3.57 (3.24 - 3.93) | 3.39 (3.08 - 3.74) |
| P for trend | <0.001 | <0.001 | <0.001 | <0.001 |

Model 1 was unadjusted;

Model 2 was adjusted for age, sex, BMI, current smoking, current drinking, SBP, DBP;

Model 3 was further adjusted for ALT, AST, TG, HDL-C, eGFR, UA, FPG;

Model 4 was additionally adjusted for DM, dyslipidemia, CHD, nephrolithiasis, hyperuricemia, lipid-lowering drugs, antidiabetic agents, diuretics, beta-blockers, calcium channel blockers, and ACEI/ARBs.

Abbreviations: WC, waist circumference; BMI, body mass index; TyG, triglyceride-glucose; METS-IR, metabolic score for insulin resistance; METS-VF, metabolic score for visceral fat; OR, odds ratio; CI, confidence interval; Other abbreviations, see Table 1.

Table S7. Sensitivity analysis of the associations between metabolic indices and large SRCs was performed after excluding participants with BMI ≥ 30kg/m^2^.

| Variables | Model 1 | Model 2 | Model 3 | Model 4 |
| --- | --- | --- | --- | --- |
|  | OR (95% CI) | OR (95% CI) | OR (95% CI) | OR (95% CI) |
| **TG/HDL-C** |  |  |  |  |
| Per SD increase | 1.24 (1.20 - 1.28) | 1.17 (1.12 - 1.21) | 1.16 (1.11 - 1.20) | 1.15 (1.11 - 1.20) |
| Quartiles |  |  |  |  |
| Q1 | Reference | Reference | Reference | Reference |
| Q2 | 1.22 (1.04 - 1.42) | 1.11 (0.95 - 1.30) | 1.08 (0.92 - 1.26) | 1.08 (0.92 - 1.26) |
| Q3 | 1.59 (1.37 - 1.85) | 1.40 (1.20 - 1.63) | 1.31 (1.13 - 1.53) | 1.30 (1.11 - 1.51) |
| Q4 | 1.85 (1.60 - 2.14) | 1.59 (1.37 - 1.85) | 1.45 (1.24 - 1.70) | 1.36 (1.15 - 1.59) |
| P for trend | <0.001 | <0.001 | <0.001 | <0.001 |
| **TyG** |  |  |  |  |
| Per SD increase | 1.30 (1.25 - 1.37) | 1.22 (1.17 - 1.28) | 1.20 (1.14 - 1.27) | 1.18 (1.11 - 1.24) |
| Quartiles |  |  |  |  |
| Q1 | Reference | Reference | Reference | Reference |
| Q2 | 1.42 (1.21 - 1.66) | 1.30 (1.11 - 1.51) | 1.24 (1.06 - 1.45) | 1.23 (1.05 - 1.44) |
| Q3 | 1.53 (1.31 - 1.78) | 1.36 (1.16 - 1.59) | 1.26 (1.08 - 1.48) | 1.25 (1.06 - 1.47) |
| Q4 | 1.96 (1.69 - 2.27) | 1.62 (1.39 - 1.88) | 1.46 (1.25 - 1.71) | 1.41 (1.20 - 1.66) |
| P for trend | <0.001 | <0.001 | <0.001 | <0.001 |
| **TyG-WC** |  |  |  |  |
| Per SD increase | 1.37 (1.28 - 1.46) | 1.25 (1.17 - 1.34) | 1.24 (1.16 - 1.33) | 1.23 (1.16 - 1.32) |
| Quartiles |  |  |  |  |
| Q1 | Reference | Reference | Reference | Reference |
| Q2 | 1.73 (1.44 - 2.07) | 1.68 (1.40 - 2.02) | 1.62 (1.35 - 1.94) | 1.62 (1.35 - 1.94) |
| Q3 | 2.77 (2.34 - 3.28) | 2.64 (2.23 - 3.13) | 2.50 (2.10 - 2.97) | 2.49 (2.10 - 2.96) |
| Q4 | 3.42 (2.90 - 4.04) | 3.19 (2.70 - 3.76) | 2.84 (2.38 - 3.38) | 2.84 (2.38 - 3.38) |
| P for trend | <0.001 | <0.001 | <0.001 | <0.001 |
| **TyG-BMI** |  |  |  |  |
| Per SD increase | 1.77 (1.67 - 1.88) | 1.43 (1.25 - 1.64) | 1.38 (1.20 - 1.58) | 1.38 (1.18 - 1.61) |
| Quartiles |  |  |  |  |
| Q1 | Reference | Reference | Reference | Reference |
| Q2 | 2.74 (2.28 - 3.30) | 2.51 (2.08 - 3.02) | 2.31 (1.91 - 2.78) | 2.31 (1.91 - 2.79) |
| Q3 | 3.05 (2.54 - 3.65) | 2.77 (2.31 - 3.33) | 2.47 (2.04 - 3.00) | 2.49 (2.06 - 3.02) |
| Q4 | 5.05 (4.22 - 6.03) | 4.79 (3.99 - 5.75) | 4.14 (3.39 - 5.07) | 4.20 (3.43 - 5.15) |
| P for trend | <0.001 | <0.001 | <0.001 | <0.001 |
| **METS-IR** |  |  |  |  |
| Per SD increase | 1.82 (1.72 - 1.93) | 1.62 (1.46 - 1.80) | 1.61 (1.44 - 1.79) | 1.63 (1.46 - 1.83) |
| Quartiles |  |  |  |  |
| Q1 | Reference | Reference | Reference | Reference |
| Q2 | 1.36 (1.11 - 1.67) | 1.25 (1.01 - 1.54) | 1.25 (1.01 - 1.55) | 1.26 (1.01 - 1.56) |
| Q3 | 4.53 (3.80 - 5.39) | 4.23 (3.54 - 5.06) | 4.35 (3.56 - 5.31) | 4.42 (3.61 - 5.40) |
| Q4 | 5.13 (4.29 - 6.13) | 4.92 (4.09 - 5.92) | 5.17 (4.12 - 6.48) | 5.28 (4.20 - 6.64) |
| P for trend | <0.001 | <0.001 | <0.001 | <0.001 |
| **METS-VF** |  |  |  |  |
| Per SD increase | 1.86 (1.73 - 1.99) | 1.70 (1.57 - 1.84) | 1.71 (1.58 - 1.85) | 1.71 (1.58 - 1.85) |
| Quartiles |  |  |  |  |
| Q1 | Reference | Reference | Reference | Reference |
| Q2 | 1.30 (1.03 - 1.65) | 1.29 (1.02 - 1.62) | 1.34 (1.06 - 1.69) | 1.34 (1.06 - 1.69) |
| Q3 | 4.21 (3.45 - 5.12) | 3.97 (3.25 - 4.84) | 4.17 (3.41 - 5.09) | 4.16 (3.41 - 5.09) |
| Q4 | 6.47 (5.35 - 7.83) | 5.33 (4.34 - 6.53) | 5.71 (4.63 - 7.04) | 5.72 (4.64 - 7.05) |
| P for trend | <0.001 | <0.001 | <0.001 | <0.001 |

Model 1 was unadjusted;

Model 2 was adjusted for age, sex, BMI, current smoking, current drinking, SBP, DBP;

Model 3 was further adjusted for ALT, AST, TG, HDL-C, eGFR, UA, FPG;

Model 4 was additionally adjusted for DM, dyslipidemia, CHD, nephrolithiasis, hyperuricemia, lipid-lowering drugs, antidiabetic agents, diuretics, beta-blockers, calcium channel blockers, and ACEI/ARBs.

Abbreviations: WC, waist circumference; BMI, body mass index; TyG, triglyceride-glucose; METS-IR, metabolic score for insulin resistance; METS-VF, metabolic score for visceral fat; OR, odds ratio; CI, confidence interval; Other abbreviations, see Table 1.

Table S8. Sensitivity analysis of the associations between metabolic indices and multiple SRCs was performed after excluding participants with BMI ≥ 30 kg/m^2^.

| Variables | Model 1 | Model 2 | Model 3 | Model 4 |
| --- | --- | --- | --- | --- |
|  | OR (95% CI) | OR (95% CI) | OR (95% CI) | OR (95% CI) |
| **TG/HDL-C** |  |  |  |  |
| Per SD increase | 1.30 (1.26 - 1.34) | 1.22 (1.17 - 1.26) | 1.18 (1.14 - 1.23) | 1.19 (1.14 - 1.23) |
| Quartiles |  |  |  |  |
| Q1 | Reference | Reference | Reference | Reference |
| Q2 | 1.26 (1.08 - 1.46) | 0.98 (0.84- 1.15) | 0.96 (0.82 - 1.12) | 0.96 (0.82 - 1.12) |
| Q3 | 1.86 (1.62 - 2.14) | 1.30 (1.12 - 1.50) | 1.24 (1.07 - 1.44) | 1.24 (1.07 - 1.44) |
| Q4 | 2.60 (2.28 - 2.97) | 1.69 (1.46 - 1.95) | 1.55 (1.33 - 1.80) | 1.55 (1.33 - 1.81) |
| P for trend | <0.001 | <0.001 | <0.001 | <0.001 |
| **TyG** |  |  |  |  |
| Per SD increase | 1.48 (1.42 - 1.54) | 1.29 (1.24 - 1.35) | 1.29 (1.23 - 1.35) | 1.26 (1.20 - 1.33) |
| Quartiles |  |  |  |  |
| Q1 | Reference | Reference | Reference | Reference |
| Q2 | 1.61 (1.38 - 1.88) | 1.32 (1.13 - 1.55) | 1.31 (1.12 - 1.54) | 1.30 (1.11 - 1.52) |
| Q3 | 3.11 (2.70 - 3.59) | 1.63 (1.41 - 1.90) | 1.62 (1.39 - 1.89) | 1.58 (1.35 - 1.84) |
| Q4 | 4.26 (3.67 - 4.96) | 2.01 (1.73 - V) | 1.98 (1.71 - 2.31) | 1.85 (1.58 - 2.17) |
| P for trend | <0.001 | <0.001 | <0.001 | <0.001 |
| **TyG-WC** |  |  |  |  |
| Per SD increase | 1.54 (1.45 - 1.63) | 1.40 (1.32 - 1.48) | 1.35 (1.28 - 1.44) | 1.32 (1.24 - 1.40) |
| Quartiles |  |  |  |  |
| Q1 |  | Reference | Reference | Reference |
| Q2 | 1.59 (1.35 - 1.87) | 1.50 (1.28 - 1.77) | 1.47 (1.25 - 1.73) | 1.46 (1.24 - 1.72) |
| Q3 | 2.22 (1.91 - 2.59) | 2.01 (1.73 - 2.35) | 1.95 (1.68 - 2.29) | 1.92 (1.64 - 2.25) |
| Q4 | 3.76 (3.26 - 4.35) | 3.63 (2.77 - 3.71) | 3.00 (2.60 - 3.49) | 2.89 (2.50 - 3.77) |
| P for trend | <0.001 | <0.001 | <0.001 | <0.001 |
| **TyG-BMI** |  |  |  |  |
| Per SD increase | 2.06 (1.95 - 2.17) | 2.11 (1.99 - 2.23) | 1.98 (1.86 - 2.10) | 1.95 (1.83 - 2.07) |
| Quartiles |  |  |  |  |
| Q1 | Reference | Reference | Reference | Reference |
| Q2 | 1.72 (1.45 - 2.04) | 1.57 (1.32 - 1.86) | 1.43 (1.20 - 1.70) | 1.40 (1.18 - 1.67) |
| Q3 | 3.03 (2.60 - 3.55) | 2.84 (2.42 - 3.24) | 2.46 (2.09 - 2.91) | 2.40 (2.04 - 2.84) |
| Q4 | 5.46 (4.69 - 6.37) | 5.45 (4.66 - 6.40) | 4.51 (3.83 - 5.34) | 4.31 (3.64 - 5.11) |
| P for trend | <0.001 | <0.001 | <0.001 | <0.001 |
| **METS-IR** |  |  |  |  |
| Per SD increase | 2.09 (1.99 - 2.20) | 2.14 (2.03 - 2.26) | 2.19 (2.04 - 2.35) | 2.14 (2.00 - 2.31) |
| Quartiles |  |  |  |  |
| Q1 | Reference | Reference | Reference | Reference |
| Q2 | 1.32 (1.09 - 1.59) | 1.21 (1.00 - 1.46) | 1.18 (0.98 - 1.43) | 1.18 (0.98 - 1.43) |
| Q3 | 3.53 (3.01 - 4.16) | 3.38 (2.87 - 4.00) | 3.33 (2.82 - 3.95) | 3.34 (2.83 - 3.96) |
| Q4 | 7.00 (7.00 - 8.21) | 7.02 (5.97 - 8.29) | 6.97 (5.90 - 8.26) | 6.99 (5.92 - 8.31) |
| P for trend | <0.001 | <0.001 | <0.001 | <0.001 |
| **METS-VF** |  |  |  |  |
| Per SD increase | 2.16 (2.02 - 2.31) | 2.24 (2.07 - 2.43) | 2.18 (1.97 - 2.41) | 2.17 (1.97 - 2.41) |
| Quartiles |  |  |  |  |
| Q1 | Reference | Reference | Reference | Reference |
| Q2 | 1.42 (1.14 - 1.77) | 1.43 (1.15 - 1.79) | 1.47 (1.18 - 1.83) | 1.60 (1.29 - 2.00) |
| Q3 | 4.41 (3.67 - 5.35) | 4.43 (3.67 - 5.37) | 4.55 (3.78 - 5.53) | 5.08 (4.20 - 6.19) |
| Q4 | 8.47 (7.10 - 10.19) | 8.10 (6.75 - 9.79) | 8.42 (7.00 - 10.19) | 8.73 (7.17 - 10.70) |
| P for trend | <0.001 | <0.001 | <0.001 | <0.001 |

Model 1 was unadjusted;

Model 2 was adjusted for age, sex, BMI, current smoking, current drinking, SBP, DBP;

Model 3 was further adjusted for ALT, AST, TG, HDL-C, eGFR, UA, FPG;

Model 4 was additionally adjusted for DM, dyslipidemia, CHD, nephrolithiasis, hyperuricemia, lipid-lowering drugs, antidiabetic agents, diuretics, beta-blockers, calcium channel blockers, and ACEI/ARBs.

Abbreviations: WC, waist circumference; BMI, body mass index; TyG, triglyceride-glucose; METS-IR, metabolic score for insulin resistance; METS-VF, metabolic score for visceral fat; OR, odds ratio; CI, confidence interval; Other abbreviations, see Table 1.

Table S9. Sensitivity analysis of the associations between metabolic indices and SRCs was performed after excluding participants with DM.

| Variables | Model 1 | Model 2 | Model 3 | Model 4 |
| --- | --- | --- | --- | --- |
|  | OR (95% CI) | OR (95% CI) | OR (95% CI) | OR (95% CI) |
| **TG/HDL-C** |  |  |  |  |
| Per SD increase | 1.27 (1.23 - 1.30) | 1.23 (1.19 - 1.26) | 1.19 (1.16 - 1.23) | 1.19 (1.16 - 1.23) |
| Quartiles |  |  |  |  |
| Q1 | Reference | Reference | Reference | Reference |
| Q2 | 1.41 (1.29 - 1.54) | 1.21 (1.11 - 1.33) | 1.18 (1.08 - 1.29) | 1.18 (1.08 - 1.29) |
| Q3 | 1.61 (1.48 - 1.76) | 1.32 (1.21 - 1.45) | 1.26 (1.15 - 1.38) | 1.24 (1.13 - 1.36) |
|  | 1.89 (1.73 - 2.06) | 1.54 (1.40 - 1.69) | 1.40 (1.27 - 1.54) | 1.38 (1.25 - 1.53) |
| P for trend | <0.001 | <0.001 | <0.001 | <0.001 |
| **TyG** |  |  |  |  |
| Per SD increase | 1.39 (1.34 - 1.43) | 1.29 (1.25 - 1.34) | 1.23 (1.19 - 1.28) | 1.23 (1.18 - 1.28) |
| Quartiles |  |  |  |  |
| Q1 | Reference | Reference | Reference | Reference |
| Q2 | 1.25 (1.15 - 1.36) | 1.09 (1.00 - 1.19) | 1.03 (0.94 - 1.13) | 1.03 (0.94 - 1.12) |
| Q3 | 1.52 (1.40 - 1.65) | 1.28 (1.17 - 1.40) | 1.18 (1.07 - 1.29) | 1.16 (1.05 - 1.27) |
|  | 2.15 (1.97 - 2.34) | 1.76 (1.61 - 1.93) | 1.54 (1.40 - 1.70) | 1.52 (1.37 - 1.68) |
| P for trend | <0.001 | <0.001 | <0.001 | <0.001 |
| **TyG-WC** |  |  |  |  |
| Per SD increase | 1.39 (1.33 - 1.45) | 1.33 (1.28 - 1.39) | 1.28 (1.23 - 1.34) | 1.28 (1.22 - 1.34) |
| Quartiles |  |  |  |  |
| Q1 | Reference | Reference | Reference | Reference |
| Q2 | 1.47 (1.34 - 1.60) | 1.43 (1.31 - 1.57) | 1.41 (1.28 - 1.54) | 1.41 (1.28 - 1.54) |
| Q3 | 1.77 (1.62 - 1.93) | 1.69 (1.54 - 1.84) | 1.64 (1.49 - 1.79) | 1.63 (1.49 - 1.78) |
| Q4 | 2.22 (2.03 - 2.42) | 2.10 (1.92 - 2.29) | 1.96 (1.79 - 2.15) | 1.95 (1.78 - 2.14) |
| P for trend | <0.001 | <0.001 | <0.001 | <0.001 |
| **TyG-BMI** |  |  |  |  |
| Per SD increase | 1.48 (1.44 - 1.53) | 1.50 (1.46 - 1.55) | 1.44 (1.39 - 1.49) | 1.44 (1.39 - 1.49) |
| Quartiles |  |  |  |  |
| Q1 | Reference | Reference | Reference | Reference |
| Q2 | 1.86 (1.69 - 2.04) | 1.74 (1.59 - 1.92) | 1.65 (1.50 - 1.82) | 1.63 (1.48 - 1.80) |
| Q3 | 2.34 (2.14 - 2.56) | 2.27 (2.06 - 2.49) | 2.08 (1.88 - 2.29) | 2.06 (1.86 - 2.27) |
| Q4 | 3.14 (2.87 - 3.43) | 3.27 (2.97 - 3.60) | 2.91 (2.63 - 3.23) | 2.89 (2.61 - 3.20) |
| P for trend | <0.001 | <0.001 | <0.001 | <0.001 |
| **METS-IR** |  |  |  |  |
| Per SD increase | 1.50 (1.46 - 1.54) | 1.53 (1.48 - 1.58) | 1.50 (1.44 - 1.56) | 1.50 (1.45 - 1.56) |
| Quartiles |  |  |  |  |
| Q1 | Reference | Reference | Reference | Reference |
| Q2 | 1.40 (1.27 - 1.54) | 1.29 (1.17 - 1.43) | 1.31 (1.18 - 1.46) | 1.30 (1.17 - 1.44) |
| Q3 | 2.55 (2.34 - 2.79) | 2.51 (2.29 - 2.76) | 2.56 (2.30 - 2.84) | 2.54 (2.28 - 2.82) |
| Q4 | 3.29 (3.01 - 3.60) | 3.46 (3.14 - 3.81) | 3.51 (3.14 - 3.94) | 3.50 (3.12 - 3.92) |
| P for trend | <0.001 | <0.001 | <0.001 | <0.001 |
| **METS-VF** |  |  |  |  |
| Per SD increase | 1.71 (1.65 - 1.78) | 1.63 (1.57 - 1.70) | 1.63 (1.57 - 1.70) | 1.61 (1.55 - 1.68) |
| Quartiles |  |  |  |  |
| Q1 | Reference | Reference | Reference | Reference |
| Q2 | 1.54 (1.40 - 1.70) | 1.54 (1.40 - 1.70) | 1.61 (1.46 - 1.78) | 1.60 (1.45 - 1.77) |
| Q3 | 2.36 (2.15 - 2.59) | 2.31 (2.10 - 2.54) | 2.40 (2.18 - 2.64) | 2.38 (2.16 - 2.61) |
| Q4 | 4.02 (3.67 - 4.40) | 3.71 (3.39 - 4.07) | 3.77 (3.43 - 4.14) | 3.66 (3.33 - 4.02) |
| P for trend | <0.001 | <0.001 | <0.001 | <0.001 |

Model 1 was unadjusted;

Model 2 was adjusted for age, sex, BMI, current smoking, current drinking, SBP, DBP;

Model 3 was further adjusted for ALT, AST, TG, HDL-C, eGFR, UA, FPG;

Model 4 was additionally adjusted for dyslipidemia, CHD, nephrolithiasis, hyperuricemia, lipid-lowering drugs, antidiabetic agents, diuretics, beta-blockers, calcium channel blockers, and ACEI/ARBs.

Abbreviations: WC, waist circumference; BMI, body mass index; TyG, triglyceride-glucose; METS-IR, metabolic score for insulin resistance; METS-VF, metabolic score for visceral fat; OR, odds ratio; CI, confidence interval; Other abbreviations, see Table 1.

Table S10. Sensitivity analysis of the associations between metabolic indices and large SRCs was performed after excluding participants with DM.

| Variables | Model 1 | Model 2 | Model 3 | Model 4 |
| --- | --- | --- | --- | --- |
|  | OR (95% CI) | OR (95% CI) | OR (95% CI) | OR (95% CI) |
| **TG/HDL-C** |  |  |  |  |
| Per SD increase | 1.24 (1.19 - 1.29) | 1.18 (1.13 - 1.24) | 1.15 (1.10 - 1.21) | 1.15 (1.10 - 1.21) |
| Quartiles |  |  |  |  |
| Q1 | Reference | Reference | Reference | Reference |
| Q2 | 1.33 (1.12 - 1.57) | 1.23 (1.04 - 1.46) | 1.18 (0.99 - 1.40) | 1.18 (0.99 - 1.40) |
| Q3 | 1.48 (1.25 - 1.74) | 1.32 (1.11 - 1.56) | 1.21 (1.02 - 1.44) | 1.20 (1.01 - 1.43) |
|  | 1.93 (1.65 - 2.27) | 1.70 (1.43 - 2.01) | 1.50 (1.26 - 1.78) | 1.48 (1.24 - 1.77) |
| P for trend | <0.001 | <0.001 | <0.001 | <0.001 |
| **TyG** |  |  |  |  |
| Per SD increase | 1.36 (1.28 - 1.44) | 1.24 (1.17 - 1.32) | 1.17 (1.10 - 1.26) | 1.17 (1.09 - 1.25) |
| Quartiles |  |  |  |  |
| Q1 | Reference | Reference | Reference | Reference |
| Q2 | 1.36 (1.15 - 1.60) | 1.27 (1.08 - 1.50) | 1.21 (1.03 - 1.43) | 1.21 (1.02 - 1.42) |
| Q3 | 1.53 (1.30 - 1.80) | 1.38 (1.17 - 1.63) | 1.27 (1.07 - 1.50) | 1.25 (1.06 - 1.48) |
|  | 2.00 (1.70 - 2.34) | 1.73 (1.47 - 2.05) | 1.52 (1.28 - 1.81) | 1.50 (1.26 - 1.79) |
| P for trend | <0.001 | <0.001 | <0.001 | <0.001 |
| **TyG-WC** |  |  |  |  |
| Per SD increase | 1.37 (1.27 - 1.47) | 1.30 (1.20 - 1.40) | 1.25 (1.16 - 1.35) | 1.25 (1.16 - 1.34) |
| Quartiles |  |  |  |  |
| Q1 | Reference | Reference | Reference | Reference |
| Q2 | 1.48 (1.22 - 1.80) | 1.42 (1.17 - 1.73) | 1.41 (1.16 - 1.72) | 1.41 (1.16 - 1.72) |
| Q3 | 2.75 (2.30 - 3.29) | 2.58 (2.16 - 3.09) | 2.54 (2.12 - 3.04) | 2.52 (2.11 - 3.02) |
| Q4 | 3.50 (2.94 - 4.18) | 3.21 (2.69 - 3.83) | 3.12 (2.61 - 3.72) | 3.11 (2.60 - 3.71) |
| P for trend | <0.001 | <0.001 | <0.001 | <0.001 |
| **TyG-BMI** |  |  |  |  |
| Per SD increase | 1.52 (1.44 - 1.60) | 1.52 (1.44 - 1.61) | 1.44 (1.35 - 1.53) | 1.44 (1.35 - 1.53) |
| Quartiles |  |  |  |  |
| Q1 | Reference | Reference | Reference | Reference |
| Q2 | 1.71 (1.41 - 2.08) | 1.59 (1.30 - 1.93) | 1.49 (1.22 - 1.81) | 1.47 (1.21 - 1.80) |
| Q3 | 2.62 (2.18 - 3.14) | 2.49 (2.06 - 3.00) | 2.24 (1.84 - 2.72) | 2.22 (1.82 - 2.70) |
| Q4 | 3.86 (3.24 - 4.61) | 3.83 (3.19 - 4.60) | 3.33 (2.73 - 4.07) | 3.32 (2.72 - 4.06) |
| P for trend | <0.001 | <0.001 | <0.001 | <0.001 |
| **METS-IR** |  |  |  |  |
| Per SD increase | 1.53 (1.45 - 1.61) | 1.53 (1.45 - 1.62) | 1.48 (1.39 - 1.57) | 1.48 (1.40 - 1.58) |
| Quartiles |  |  |  |  |
| Q1 | Reference | Reference | Reference | Reference |
| Q2 | 1.57 (1.28 - 1.93) | 1.54 (1.25 - 1.89) | 1.45 (1.18 - 1.79) | 1.44 (1.17 - 1.78) |
| Q3 | 3.21 (2.67 - 3.86) | 3.16 (2.62 - 3.81) | 2.94 (2.42 - 3.58) | 2.91 (2.39 - 3.54) |
| Q4 | 4.29 (3.58 - 5.14) | 4.24 (3.52 - 5.11) | 3.90 (3.19 - 4.77) | 3.85 (3.15 - 4.72) |
| P for trend | <0.001 | <0.001 | <0.001 | <0.001 |
| **METS-VF** |  |  |  |  |
| Per SD increase | 1.66 (1.54 - 1.79) | 1.59 (1.45 - 1.74) | 1.58 (1.44 - 1.74) | 1.58 (1.44 - 1.74) |
| Quartiles |  |  |  |  |
| Q1 | Reference | Reference | Reference | Reference |
| Q2 | 1.74 (1.40 - 2.17) | 1.75 (1.41 - 2.18) | 1.76 (1.41 - 2.19) | 1.75 (1.41 - 2.18) |
| Q3 | 3.17 (2.59 - 3.87) | 3.15 (2.57 - 3.85) | 3.11 (2.54 - 3.81) | 3.09 (2.52 - 3.78) |
| Q4 | 5.28 (4.36 - 6.40) | 5.00 (4.10 - 6.10) | 4.81 (3.93 - 5.88) | 4.75 (3.88 - 5.81) |
| P for trend | <0.001 | <0.001 | <0.001 | <0.001 |

Model 1 was unadjusted;

Model 2 was adjusted for age, sex, BMI, current smoking, current drinking, SBP, DBP;

Model 3 was further adjusted for ALT, AST, TG, HDL-C, eGFR, UA, FPG;

Model 4 was additionally adjusted for dyslipidemia, CHD, nephrolithiasis, hyperuricemia, lipid-lowering drugs, antidiabetic agents, diuretics, beta-blockers, calcium channel blockers, and ACEI/ARBs.

Abbreviations: WC, waist circumference; BMI, body mass index; TyG, triglyceride-glucose; METS-IR, metabolic score for insulin resistance; METS-VF, metabolic score for visceral fat; OR, odds ratio; CI, confidence interval; Other abbreviations, see Table 1.

Table S11. Sensitivity analysis of the associations between metabolic indices and multiple SRCs was performed after excluding participants with DM.

| Variables | Model 1 | Model 2 | Model 3 | Model 4 |
| --- | --- | --- | --- | --- |
|  | OR (95% CI) | OR (95% CI) | OR (95% CI) | OR (95% CI) |
| **TG/HDL-C** |  |  |  |  |
| Per SD increase | 1.33 (1.28 - 1.37) | 1.25 (1.20 - 1.30) | 1.23 (1.18 - 1.28) | 1.23 (1.17 - 1.28) |
| Quartiles |  |  |  |  |
| Q1 | Reference | Reference | Reference | Reference |
| Q2 | 1.47 (1.23 - 1.76) | 1.20 (1.01 - 1.44) | 1.03 (0.85 - 1.24) | 1.03 (0.85 - 1.24) |
| Q3 | 2.69 (2.29 - 3.16) | 2.04 (1.72 - 2.41) | 1.59 (1.32 - 1.91) | 1.59 (1.32 - 1.92) |
| Q4 | 3.24 (2.76 - 3.80) | 2.34 (1.98 - 2.78) | 1.65 (1.34 - 2.02) | 1.65 (1.34 - 2.04) |
| P for trend | <0.001 | <0.001 | <0.001 | <0.001 |
| **TyG** |  |  |  |  |
| Per SD increase | 1.55 (1.47 - 1.64) | 1.40 (1.32 - 1.48) | 1.29 (1.21 - 1.37) | 1.29 (1.20 - 1.38) |
| Quartiles |  |  |  |  |
| Q1 | Reference | Reference | Reference | Reference |
| Q2 | 2.31 (1.96 - 2.71) | 1.98 (1.68 - 2.34) | 1.79 (1.52 - 2.12) | 1.79 (1.51 - 2.12) |
| Q3 | 2.08 (1.76 - 2.46) | 1.62 (1.36 - 1.92) | 1.37 (1.15 - 1.64) | 1.36 (1.14 - 1.63) |
| Q4 | 3.65 (3.11 - 4.29) | 2.68 (2.27 - 3.17) | 2.11 (1.75 - 2.54) | 2.10 (1.74 - 2.54) |
| P for trend | <0.001 | <0.001 | <0.001 | <0.001 |
| **TyG-WC** |  |  |  |  |
| Per SD increase | 1.57 (1.47 - 1.68) | 1.47 (1.37 - 1.57) | 1.40 (1.31 - 1.51) | 1.39 (1.29 - 1.49) |
| Quartiles |  |  |  |  |
| Q1 | Reference | Reference | Reference | Reference |
| Q2 | 1.26 (1.03 - 1.54) | 1.19 (0.97 - 1.46) | 1.15 (0.94 - 1.41) | 1.15 (0.94 - 1.41) |
| Q3 | 3.96 (3.34 - 4.70) | 3.67 (3.08 - 4.38) | 3.45 (2.89 - 4.12) | 3.45 (2.89 - 4.11) |
| Q4 | 4.50 (3.79 - 5.34) | 3.98 (3.31 - 4.79) | 3.56 (2.95 - 4.28) | 3.56 (2.96 - 4.30) |
| P for trend | <0.001 | <0.001 | <0.001 | <0.001 |
| **TyG-BMI** |  |  |  |  |
| Per SD increase | 1.68 (1.60 - 1.76) | 1.67 (1.59 - 1.76) | 1.57 (1.49 - 1.66) | 1.56 (1.48 - 1.65) |
| Quartiles |  |  |  |  |
| Q1 | Reference | Reference | Reference | Reference |
| Q2 | 1.63 (1.35 - 1.98) | 1.47 (1.21 - 1.79) | 1.40 (1.14 - 1.73) | 1.39 (1.13 - 1.71) |
| Q3 | 2.94 (2.47 - 3.50) | 2.71 (2.27 - 3.24) | 2.56 (2.05 - 3.19) | 2.55 (2.04 - 3.18) |
| Q4 | 5.18 (4.38 - 6.12) | 5.09 (4.28 - 6.05) | 5.05 (3.79 - 6.73) | 5.04 (3.77 - 6.74) |
| P for trend | <0.001 | <0.001 | <0.001 | <0.001 |
| **METS-IR** |  |  |  |  |
| Per SD increase | 1.73 (1.65 - 1.82) | 1.73 (1.64 - 1.82) | 1.63 (1.54 - 1.73) | 1.62 (1.52 - 1.72) |
| Quartiles |  |  |  |  |
| Q1 | Reference | Reference | Reference | Reference |
| Q2 | 1.92 (1.56 - 2.36) | 1.72 (1.40 - 2.12) | 1.65 (1.33 - 2.05) | 1.64 (1.32 - 2.04) |
| Q3 | 3.89 (3.23 - 4.69) | 3.63 (2.99 - 4.40) | 3.39 (2.75 - 4.18) | 3.37 (2.73 - 4.16) |
| Q4 | 6.75 (5.64 - 8.09) | 6.63 (5.49 - 8.00) | 6.06 (4.89 - 7.53) | 6.05 (4.87 - 7.51) |
| P for trend | <0.001 | <0.001 | <0.001 | <0.001 |
| **METS-VF** |  |  |  |  |
| Per SD increase | 2.01 (1.87 - 2.16) | 1.95 (1.75 - 2.16) | 1.94 (1.75 - 2.16) | 1.93 (1.74 - 2.15) |
| Quartiles |  |  |  |  |
| Q1 | Reference | Reference | Reference | Reference |
| Q2 | 1.72 (1.37 - 2.16) | 1.73 (1.38 - 2.17) | 1.80 (1.43 - 2.26) | 1.79 (1.42 - 2.24) |
| Q3 | 3.98 (3.25 - 4.88) | 3.97 (3.24 - 4.87) | 4.05 (3.29 - 4.99) | 4.01 (3.25 - 4.93) |
| Q4 | 8.25 (6.81 - 10.01) | 7.83 (6.42 - 9.55) | 7.63 (6.16 - 9.45) | 7.37 (5.95 - 9.13) |
| P for trend | <0.001 | <0.001 | <0.001 | <0.001 |

Model 1 was unadjusted;

Model 2 was adjusted for age, sex, BMI, current smoking, current drinking, SBP, DBP;

Model 3 was further adjusted for ALT, AST, TG, HDL-C, eGFR, UA, FPG;

Model 4 was additionally adjusted for dyslipidemia, CHD, nephrolithiasis, hyperuricemia, lipid-lowering drugs, antidiabetic agents, diuretics, beta-blockers, calcium channel blockers, and ACEI/ARBs.

Abbreviations: WC, waist circumference; BMI, body mass index; TyG, triglyceride-glucose; METS-IR, metabolic score for insulin resistance; METS-VF, metabolic score for visceral fat; OR, odds ratio; CI, confidence interval; Other abbreviations, see Table 1.

Table S12. Sensitivity analysis of the associations between metabolic indices and SRCs was performed after excluding participants with dyslipidemia.

| Variables | Model 1 | Model 2 | Model 3 | Model 4 |
| --- | --- | --- | --- | --- |
|  | OR (95% CI) | OR (95% CI) | OR (95% CI) | OR (95% CI) |
| **TG/HDL-C** |  |  |  |  |
| Per SD increase | 1.19 (1.16 - 1.22) | 1.18 (1.15 - 1.21) | 1.14 (1.11 - 1.18) | 1.13 (1.10 - 1.17) |
| Quartiles |  |  |  |  |
| Q1 | Reference | Reference | Reference | Reference |
| Q2 | 1.19 (1.11 - 1.29) | 1.14 (1.06 - 1.23) | 1.13 (1.04 - 1.22) | 1.08 (1.00 - 1.17) |
| Q3 | 1.39 (1.29 - 1.50) | 1.29 (1.19 - 1.40) | 1.25 (1.16 - 1.36) | 1.19 (1.09 - 1.29) |
| Q4 | 1.63 (1.50 - 1.76) | 1.46 (1.35 - 1.58) | 1.40 (1.28 - 1.52) | 1.29 (1.18 - 1.40) |
| P for trend | <0.001 | <0.001 | <0.001 | <0.001 |
| **TyG** |  |  |  |  |
| Per SD increase | 1.26 (1.23 - 1.29) | 1.21 (1.18 - 1.25) | 1.19 (1.16 - 1.23) | 1.15 (1.12 - 1.19) |
| Quartiles |  |  |  |  |
| Q1 | Reference | Reference | Reference | Reference |
| Q2 | 1.21 (1.12 - 1.31) | 1.17 (1.08 - 1.26) | 1.14 (1.06 - 1.23) | 1.11 (1.03 - 1.20) |
| Q3 | 1.37 (1.27 - 1.48) | 1.28 (1.18 - 1.38) | 1.24 (1.14 - 1.34) | 1.17 (1.08 - 1.27) |
| Q4 | 1.69 (1.56 - 1.82) | 1.52 (1.41 - 1.65) | 1.45 (1.33 - 1.57) | 1.31 (1.20 - 1.43) |
| P for trend | <0.001 | <0.001 | <0.001 | <0.001 |
| **TyG-WC** |  |  |  |  |
| Per SD increase | 1.30 (1.25 - 1.35) | 1.22 (1.17 - 1.27) | 1.20 (1.15 - 1.24) | 1.20 (1.15 - 1.25) |
| Quartiles |  |  |  |  |
| Q1 | Reference | Reference | Reference | Reference |
| Q2 | 1.28 (1.18 - 1.38) | 1.22 (1.12 - 1.33) | 1.21 (1.11 - 1.32) | 1.22 (1.12 - 1.32) |
| Q3 | 1.51 (1.39 - 1.63) | 1.39 (1.27 - 1.51) | 1.36 (1.25 - 1.48) | 1.36 (1.25 - 1.49) |
| Q4 | 1.87 (1.73 - 2.02) | 1.66 (1.51 - 1.82) | 1.59 (1.44 - 1.75) | 1.60 (1.45 - 1.76) |
| P for trend | <0.001 | <0.001 | <0.001 | <0.001 |
| **TyG-BMI** |  |  |  |  |
| Per SD increase | 1.37 (1.33 - 1.40) | 1.34 (1.30 - 1.37) | 1.32 (1.22 - 1.43) | 1.30 (1.19 - 1.42) |
| Quartiles |  |  |  |  |
| Q1 | Reference | Reference | Reference | Reference |
| Q2 | 1.37 (1.26 - 1.48) | 1.23 (1.13 - 1.33) | 1.23 (1.12 - 1.35) | 1.22 (1.11 - 1.34) |
| Q3 | 1.90 (1.75 - 2.06) | 1.74 (1.60 - 1.89) | 1.74 (1.57 - 1.94) | 1.73 (1.55 - 1.93) |
| Q4 | 2.52 (2.33 - 2.73) | 2.41 (2.22 - 2.62) | 2.45 (2.11 - 2.84) | 2.42 (2.07 - 2.82) |
| P for trend | <0.001 | <0.001 | <0.001 | <0.001 |
| **METS-IR** |  |  |  |  |
| Per SD increase | 1.38 (1.34 - 1.42) | 1.40 (1.37 - 1.45) | 1.37 (1.33 - 1.41) | 1.36 (1.32 - 1.41) |
| Quartiles |  |  |  |  |
| Q1 | Reference | Reference | Reference | Reference |
| Q2 | 1.52 (1.40 - 1.65) | 1.45 (1.33 - 1.58) | 1.41 (1.30 - 1.54) | 1.40 (1.28 - 1.52) |
| Q3 | 2.00 (1.84 - 2.17) | 1.96 (1.80 - 2.14) | 1.87 (1.72 - 2.04) | 1.85 (1.70 - 2.02) |
| Q4 | 2.67 (2.47 - 2.89) | 2.77 (2.54 - 3.01) | 2.61 (2.39 - 2.84) | 2.56 (2.34 - 2.80) |
| P for trend | <0.001 | <0.001 | <0.001 | <0.001 |
| **METS-VF** |  |  |  |  |
| Per SD increase | 1.51 (1.45 - 1.56) | 1.53 (1.46 - 1.59) | 1.50 (1.44 - 1.56) | 1.47 (1.41 - 1.53) |
| Quartiles |  |  |  |  |
| Q1 | Reference | Reference | Reference | Reference |
| Q2 | 1.37 (1.25 - 1.49) | 1.51 (1.38 - 1.65) | 1.51 (1.38 - 1.65) | 1.50 (1.38 - 1.64) |
| Q3 | 2.10 (1.93 - 2.28) | 2.32 (2.13 - 2.53) | 2.29 (2.10 - 2.50) | 2.27 (2.08 - 2.47) |
| Q4 | 2.81 (2.59 - 3.04) | 2.79 (2.55 - 3.06) | 2.71 (2.47 - 2.96) | 2.60 (2.37 - 2.85) |
| P for trend | <0.001 | <0.001 | <0.001 | <0.001 |

Model 1 was unadjusted;

Model 2 was adjusted for age, sex, BMI, current smoking, current drinking, SBP, DBP;

Model 3 was further adjusted for ALT, AST, TG, HDL-C, eGFR, UA, FPG;

Model 4 was additionally adjusted for DM, CHD, nephrolithiasis, hyperuricemia, lipid-lowering drugs, antidiabetic agents, diuretics, beta-blockers, calcium channel blockers, and ACEI/ARBs.

Abbreviations: WC, waist circumference; BMI, body mass index; TyG, triglyceride-glucose; METS-IR, metabolic score for insulin resistance; METS-VF, metabolic score for visceral fat; OR, odds ratio; CI, confidence interval; Other abbreviations, see Table 1.

Table S13. Sensitivity analysis of the associations between metabolic indices and large SRCs was performed after excluding participants with dyslipidemia.

| Variables | Model 1 | Model 2 | Model 3 | Model 4 |
| --- | --- | --- | --- | --- |
|  | OR (95% CI) | OR (95% CI) | OR (95% CI) | OR (95% CI) |
| **TG/HDL-C** |  |  |  |  |
| Per SD increase | 1.27 (1.22 - 1.32) | 1.21 (1.15 - 1.26) | 1.15 (1.09 - 1.21) | 1.15 (1.09 - 1.20) |
| Quartiles |  |  |  |  |
| Q1 | Reference | Reference | Reference | Reference |
| Q2 | 1.74 (1.46 - 2.08) | 1.48 (1.23 - 1.77) | 1.40 (1.17 - 1.67) | 1.40 (1.17 - 1.68) |
| Q3 | 2.07 (1.74 - 2.48) | 1.63 (1.36 - 1.95) | 1.46 (1.22 - 1.76) | 1.46 (1.22 - 1.76) |
| Q4 | 2.62 (2.21 - 3.12) | 1.96 (1.63 - 2.35) | 1.64 (1.36 - 1.98) | 1.64 (1.35 - 1.98) |
| P for trend | <0.001 | <0.001 | <0.001 | <0.001 |
| **TyG** |  |  |  |  |
| Per SD increase | 1.46 (1.39 - 1.54) | 1.33 (1.26 - 1.40) | 1.20 (1.12 - 1.28) | 1.19 (1.11 - 1.27) |
| Quartiles |  |  |  |  |
| Q1 | Reference | Reference | Reference | Reference |
| Q2 | 1.59 (1.34 - 1.89) | 1.53 (1.28 - 1.82) | 1.47 (1.23 - 1.75) | 1.46 (1.22 - 1.74) |
| Q3 | 1.76 (1.47 - 2.10) | 1.61 (1.34 - 1.92) | 1.51 (1.26 - 1.81) | 1.50 (1.25 - 1.79) |
| Q4 | 2.75 (2.33 - 3.26) | 2.39 (2.01 - 2.84) | 2.22 (1.86 - 2.65) | 2.19 (1.83 - 2.61) |
| P for trend | <0.001 | <0.001 | <0.001 | <0.001 |
| **TyG-WC** |  |  |  |  |
| Per SD increase | 1.49 (1.39 - 1.61) | 1.39 (1.29 - 1.50) | 1.28 (1.19 - 1.38) | 1.28 (1.19 - 1.39) |
| Quartiles |  |  |  |  |
| Q1 | Reference | Reference | Reference | Reference |
| Q2 | 1.63 (1.29 - 2.06) | 1.56 (1.24 - 1.97) | 1.52 (1.20 - 1.92) | 1.52 (1.21 - 1.92) |
| Q3 | 4.19 (3.42 - 5.14) | 3.91 (3.18 - 4.80) | 3.69 (3.00 - 4.53) | 3.69 (3.00 - 4.54) |
| Q4 | 5.37 (4.39 - 6.56) | 4.75 (3.88 - 5.81) | 4.23 (3.45 - 5.20) | 4.24 (3.45 - 5.21) |
| P for trend | <0.001 | <0.001 | <0.001 | <0.001 |
| **TyG-BMI** |  |  |  |  |
| Per SD increase | 1.59 (1.51 - 1.67) | 1.59 (1.51 - 1.67) | 1.47 (1.38 - 1.55) | 1.48 (1.39 - 1.56) |
| Quartiles |  |  |  |  |
| Q1 | Reference | Reference | Reference | Reference |
| Q2 | 2.02 (1.63 - 2.51) | 1.86 (1.49 - 2.31) | 1.69 (1.35 - 2.11) | 1.68 (1.34 - 2.09) |
| Q3 | 3.18 (2.59 - 3.91) | 2.98 (2.41 - 3.67) | 2.53 (2.04 - 3.14) | 2.53 (2.04 - 3.15) |
| Q4 | 5.61 (4.62 - 6.82) | 5.44 (4.46 - 6.65) | 4.37 (3.53 - 5.40) | 4.40 (3.56 - 5.44) |
| P for trend | <0.001 | <0.001 | <0.001 | <0.001 |
| **METS-IR** |  |  |  |  |
| Per SD increase | 1.61 (1.53 - 1.70) | 1.62 (1.53 - 1.70) | 1.51 (1.41 - 1.61) | 1.52 (1.42 - 1.62) |
| Quartiles |  |  |  |  |
| Q1 | Reference | Reference | Reference | Reference |
| Q2 | 2.27 (1.81 - 2.85) | 2.12 (1.69 - 2.67) | 2.09 (1.65 - 2.65) | 2.09 (1.65 - 2.65) |
| Q3 | 3.63 (2.93 - 4.50) | 3.45 (2.77 - 4.30) | 3.33 (2.63 - 4.22) | 3.35 (2.64 - 4.24) |
| Q4 | 6.23 (5.08 - 7.65) | 6.12 (4.95 - 7.56) | 5.73 (4.50 - 7.30) | 5.81 (4.55 - 7.40) |
| P for trend | <0.001 | <0.001 | <0.001 | <0.001 |
| **METS-VF** |  |  |  |  |
| Per SD increase | 1.86 (1.72 - 2.02) | 1.66 (1.51 - 1.84) | 1.69 (1.53 - 1.86) | 1.69 (1.53 - 1.87) |
| Quartiles |  |  |  |  |
| Q1 | Reference | Reference | Reference | Reference |
| Q2 | 1.91 (1.49 - 2.46) | 1.93 (1.50 - 2.48) | 1.97 (1.53 - 2.53) | 1.97 (1.53 - 2.53) |
| Q3 | 3.78 (3.00 - 4.76) | 3.80 (3.01 - 4.81) | 3.85 (3.04 - 4.86) | 3.86 (3.05 - 4.88) |
| Q4 | 7.47 (6.00 - 9.29) | 7.22 (5.69 - 9.17) | 7.32 (5.76 - 9.29) | 7.34 (5.78 - 9.33) |
| P for trend | <0.001 | <0.001 | <0.001 | <0.001 |

Model 1 was unadjusted;

Model 2 was adjusted for age, sex, BMI, current smoking, current drinking, SBP, DBP;

Model 3 was further adjusted for ALT, AST, TG, HDL-C, eGFR, UA, FPG;

Model 4 was additionally adjusted for DM, CHD, nephrolithiasis, hyperuricemia, lipid-lowering drugs, antidiabetic agents, diuretics, beta-blockers, calcium channel blockers, and ACEI/ARBs.

Abbreviations: WC, waist circumference; BMI, body mass index; TyG, triglyceride-glucose; METS-IR, metabolic score for insulin resistance; METS-VF, metabolic score for visceral fat; OR, odds ratio; CI, confidence interval; Other abbreviations, see Table 1.

Table S14. Sensitivity analysis of the associations between metabolic indices and multiple SRCs was performed after excluding participants with dyslipidemia.

| Variables | Model 1 | Model 2 | Model 3 | Model 4 |
| --- | --- | --- | --- | --- |
|  | OR (95% CI) | OR (95% CI) | OR (95% CI) | OR (95% CI) |
| **TG/HDL-C** |  |  |  |  |
| Per SD increase | 1.30 (1.26 - 1.35) | 1.24 (1.19 - 1.29) | 1.19 (1.14 - 1.25) | 1.20 (1.15 - 1.25) |
| Quartiles |  |  |  |  |
| Q1 | Reference | Reference | Reference | Reference |
| Q2 | 1.39 (1.18 - 1.63) | 1.16 (0.98 - 1.36) | 1.11 (0.94 - 1.31) | 1.11 (0.94 - 1.31) |
| Q3 | 1.94 (1.67 - 2.27) | 1.48 (1.26 - 1.74) | 1.38 (1.17 - 1.62) | 1.38 (1.18 - 1.63) |
| Q4 | 2.87 (2.48 - 3.33) | 2.09 (1.78 - 2.45) | 1.85 (1.57 - 2.17) | 1.85 (1.57 - 2.19) |
| P for trend | <0.001 | <0.001 | <0.001 | <0.001 |
| **TyG** |  |  |  |  |
| Per SD increase | 1.51 (1.45 - 1.59) | 1.36 (1.30 - 1.43) | 1.23 (1.15 - 1.32) | 1.24 (1.16 - 1.32) |
| Quartiles |  |  |  |  |
| Q1 | Reference | Reference | Reference | Reference |
| Q2 | 1.91 (1.62 - 2.25) | 1.64 (1.39 - 1.94) | 1.51 (1.27 - 1.78) | 1.50 (1.27 - 1.78) |
| Q3 | 2.41 (2.05 - 2.84) | 1.87 (1.58 - 2.21) | 1.61 (1.36 - 1.91) | 1.61 (1.35 - 1.91) |
| Q4 | 3.53 (3.02 - 4.13) | 2.50 (2.12 - 2.94) | 1.91 (1.60 - 2.28) | 1.91 (1.60 - 2.29) |
| P for trend | <0.001 | <0.001 | <0.001 | <0.001 |
| **TyG-WC** |  |  |  |  |
| Per SD increase | 1.55 (1.46 - 1.66) | 1.44 (1.35 - 1.54) | 1.27 (1.18 - 1.37) | 1.27 (1.18 - 1.37) |
| Quartiles |  |  |  |  |
| Q1 | Reference | Reference | Reference | Reference |
| Q2 | 1.48 (1.23 - 1.77) | 1.42 (1.18 - 1.70) | 1.37 (1.14 - 1.65) | 1.38 (1.15 - 1.66) |
| Q3 | 2.42 (2.04 - 2.86) | 2.23 (1.88 - 2.65) | 2.10 (1.77 - 2.49) | 2.10 (1.77 - 2.50) |
| Q4 | 4.18 (3.56 - 4.90) | 3.71 (3.15 - 4.35) | 3.25 (2.74 - 3.86) | 3.25 (2.74 - 3.86) |
| P for trend | <0.001 | <0.001 | <0.001 | <0.001 |
| **TyG-BMI** |  |  |  |  |
| Per SD increase | 1.61 (1.54 - 1.68) | 1.59 (1.52 - 1.67) | 1.51 (1.44 - 1.59) | 1.52 (1.44 - 1.59) |
| Quartiles |  |  |  |  |
| Q1 | Reference | Reference | Reference | Reference |
| Q2 | 1.47 (1.21 - 1.79) | 1.32 (1.09 - 1.61) | 1.22 (1.00 - 1.48) | 1.21 (0.99 - 1.47) |
| Q3 | 3.47 (2.92 - 4.12) | 3.23 (2.71 - 3.86) | 2.84 (2.37 - 3.41) | 2.84 (2.38 - 3.42) |
| Q4 | 4.81 (4.08 - 5.69) | 4.66 (3.93 - 5.55) | 3.97 (3.33 - 4.77) | 3.99 (3.34 - 4.79) |
| P for trend | <0.001 | <0.001 | <0.001 | <0.001 |
| **METS-IR** |  |  |  |  |
| Per SD increase | 1.67 (1.59 - 1.74) | 1.64 (1.57 - 1.73) | 1.58 (1.49 - 1.67) | 1.59 (1.50 - 1.68) |
| Quartiles |  |  |  |  |
| Q1 | Reference | Reference | Reference | Reference |
| Q2 | 1.41 (1.09 - 1.59) | 1.35 (1.10 - 1.67) | 1.33 (1.08 - 1.65) | 1.31 (1.06 - 1.62) |
| Q3 | 3.33 (2.79 - 3.99) | 3.21 (2.68 - 3.86) | 3.13 (2.57 - 3.83) | 3.08 (2.53 - 3.76) |
| Q4 | 5.82 (4.92 - 6.92) | 5.60 (4.72 - 6.69) | 5.46 (4.48 - 6.70) | 5.36 (4.39 - 6.58) |
| P for trend | <0.001 | <0.001 | <0.001 | <0.001 |
| **METS-VF** |  |  |  |  |
| Per SD increase | 2.15 (2.00 - 2.31) | 1.92 (1.74 - 2.11) | 1.93 (1.74 - 2.14) | 1.93 (1.74 - 2.14) |
| Quartiles |  |  |  |  |
| Q1 | Reference | Reference | Reference | Reference |
| Q2 | 1.41 (1.12 - 1.77) | 1.40 (1.11 - 1.77) | 1.45 (1.15 - 1.83) | 1.45 (1.15 - 1.84) |
| Q3 | 3.71 (3.03 - 4.54) | 3.58 (2.92 - 4.39) | 3.72 (3.03 - 4.57) | 3.72 (3.03 - 4.58) |
| Q4 | 8.07 (6.67 - 9.76) | 7.11 (5.79 - 8.73) | 7.22 (5.86 - 8.90) | 7.19 (5.84 - 8.86) |
| P for trend | <0.001 | <0.001 | <0.001 | <0.001 |

Model 1 was unadjusted;

Model 2 was adjusted for age, sex, BMI, current smoking, current drinking, SBP, DBP;

Model 3 was further adjusted for ALT, AST, TG, HDL-C, eGFR, UA, FPG;

Model 4 was additionally adjusted for DM, CHD, nephrolithiasis, hyperuricemia, lipid-lowering drugs, antidiabetic agents, diuretics, beta-blockers, calcium channel blockers, and ACEI/ARBs.

Abbreviations: WC, waist circumference; BMI, body mass index; TyG, triglyceride-glucose; METS-IR, metabolic score for insulin resistance; METS-VF, metabolic score for visceral fat; OR, odds ratio; CI, confidence interval; Other abbreviations, see Table 1.

Table S15. Sensitivity analysis of the associations between metabolic indices and SRCs was performed after excluding participants with nephrolithiasis.

| Variables | Model 1 | Model 2 | Model 3 | Model 4 |
| --- | --- | --- | --- | --- |
|  | OR (95% CI) | OR (95% CI) | OR (95% CI) | OR (95% CI) |
| **TG/HDL-C** |  |  |  |  |
| Per SD increase | 1.18 (1.15 - 1.21) | 1.11 (1.08 - 1.14) | 1.09 (1.06 - 1.12) | 1.08 (1.05 - 1.12) |
| Quartiles |  |  |  |  |
| Q1 | Reference | Reference | Reference | Reference |
| Q2 | 1.29 (1.19 - 1.39) | 1.24 (1.15 - 1.34) | 1.23 (1.14 - 1.33) | 1.14 (1.06 - 1.24) |
| Q3 | 1.42 (1.31 - 1.53) | 1.32 (1.22 - 1.43) | 1.30 (1.20 - 1.41) | 1.16 (1.07 - 1.26) |
| Q4 | 1.72 (1.59 - 1.85) | 1.55 (1.44 - 1.68) | 1.52 (1.40 - 1.65) | 1.33 (1.22 - 1.45) |
| P for trend | <0.001 | <0.001 | <0.001 | <0.001 |
| **TyG** |  |  |  |  |
| Per SD increase | 1.28 (1.25 - 1.31) | 1.23 (1.20 - 1.27) | 1.15 (1.12 - 1.19) | 1.14 (1.11 - 1.18) |
| Quartiles |  |  |  |  |
| Q1 | Reference | Reference | Reference | Reference |
| Q2 | 1.29 (1.19 - 1.40) | 1.20 (1.11 - 1.30) | 1.19 (1.10 - 1.28) | 1.20 (1.10 - 1.29) |
| Q3 | 1.45 (1.34 - 1.57) | 1.32 (1.22 - 1.43) | 1.25 (1.16 - 1.36) | 1.25 (1.15 - 1.35) |
| Q4 | 1.83 (1.70 - 1.97) | 1.63 (1.51 - 1.77) | 1.47 (1.36 - 1.60) | 1.40 (1.29 - 1.53) |
| P for trend | <0.001 | <0.001 | <0.001 | <0.001 |
| **TyG-WC** |  |  |  |  |
| Per SD increase | 1.37 (1.32 - 1.42) | 1.29 (1.24 - 1.34) | 1.26 (1.21 - 1.31) | 1.25 (1.21 - 1.30) |
| Quartiles |  |  |  |  |
| Q1 | Reference | Reference | Reference | Reference |
| Q2 | 1.23 (1.14 - 1.33) | 1.18 (1.09 - 1.28) | 1.17 (1.08 - 1.27) | 1.16 (1.07 - 1.25) |
| Q3 | 1.42 (1.32 - 1.53) | 1.31 (1.21 - 1.42) | 1.29 (1.19 - 1.40) | 1.27 (1.17 - 1.37) |
| Q4 | 1.86 (1.72 - 2.00) | 1.67 (1.55 - 1.81) | 1.63 (1.51 - 1.77) | 1.54 (1.43 - 1.67) |
| P for trend | <0.001 | <0.001 | <0.001 | <0.001 |
| **TyG-BMI** |  |  |  |  |
| Per SD increase | 1.42 (1.39 - 1.46) | 1.41 (1.37 - 1.45) | 1.38 (1.33 - 1.42) | 1.37 (1.33 - 1.41) |
| Quartiles |  |  |  |  |
| Q1 | Reference | Reference | Reference | Reference |
| Q2 | 1.46 (1.34 - 1.58) | 1.33 (1.22 - 1.45) | 1.31 (1.20 - 1.42) | 1.29 (1.18 - 1.41) |
| Q3 | 1.97 (1.82 - 2.13) | 1.83 (1.68 - 1.99) | 1.78 (1.63 - 1.94) | 1.74 (1.59 - 1.90) |
| Q4 | 2.88 (2.66 - 3.11) | 2.83 (2.60 - 3.07) | 2.73 (2.50 - 2.98) | 2.64 (2.41 - 2.88) |
| P for trend | <0.001 | <0.001 | <0.001 | <0.001 |
| **METS-IR** |  |  |  |  |
| Per SD increase | 1.43 (1.39 - 1.47) | 1.41 (1.37 - 1.45) | 1.43 (1.38 - 1.48) | 1.42 (1.37 - 1.47) |
| Quartiles |  |  |  |  |
| Q1 | Reference | Reference | Reference | Reference |
| Q2 | 1.58 (1.45 - 1.72) | 1.53 (1.41 - 1.67) | 1.51 (1.38 - 1.64) | 1.47 (1.35 - 1.60) |
| Q3 | 2.15 (1.99 - 2.34) | 2.07 (1.91 - 2.25) | 2.02 (1.86 - 2.20) | 1.96 (1.79 - 2.13) |
| Q4 | 3.07 (2.83 - 3.32) | 2.94 (2.71 - 3.19) | 2.84 (2.61 - 3.10) | 2.74 (2.51 - 2.99) |
| P for trend | <0.001 | <0.001 | <0.001 | <0.001 |
| **METS-VF** |  |  |  |  |
| Per SD increase | 1.59 (1.54 - 1.65) | 1.54 (1.48 - 1.60) | 1.52 (1.47 - 1.58) | 1.50 (1.44 - 1.56) |
| Quartiles |  |  |  |  |
| Q1 | Reference | Reference | Reference | Reference |
| Q2 | 1.57 (1.44 - 1.70) | 1.59 (1.46 - 1.73) | 1.62 (1.49 - 1.76) | 1.62 (1.49 - 1.76) |
| Q3 | 2.07 (1.91 - 2.24) | 2.07 (1.91 - 2.25) | 2.07 (1.90 - 2.25) | 2.05 (1.89 - 2.23) |
| Q4 | 3.17 (2.93 - 3.43) | 2.99 (2.76 - 3.25) | 2.91 (2.68 - 3.16) | 2.84 (2.61 - 3.08) |
| P for trend | <0.001 | <0.001 | <0.001 | <0.001 |

Model 1 was unadjusted;

Model 2 was adjusted for age, sex, BMI, current smoking, current drinking, SBP, DBP;

Model 3 was further adjusted for ALT, AST, TG, HDL-C, eGFR, UA, FPG;

Model 4 was additionally adjusted for DM, dyslipidemia, CHD, hyperuricemia, lipid-lowering drugs, antidiabetic agents, diuretics, beta-blockers, calcium channel blockers, and ACEI/ARBs.

Abbreviations: WC, waist circumference; BMI, body mass index; TyG, triglyceride-glucose; METS-IR, metabolic score for insulin resistance; METS-VF, metabolic score for visceral fat; OR, odds ratio; CI, confidence interval; Other abbreviations, see Table 1.

Table S16. Sensitivity analysis of the associations between metabolic indices and large SRCs was performed after excluding participants with nephrolithiasis.

| Variables | Model 1 | Model 2 | Model 3 | Model 4 |
| --- | --- | --- | --- | --- |
|  | OR (95% CI) | OR (95% CI) | OR (95% CI) | OR (95% CI) |
| **TG/HDL-C** |  |  |  |  |
| Per SD increase | 1.24 (1.19 - 1.28) | 1.18 (1.14 - 1.23) | 1.15 (1.10 - 1.20) | 1.15 (1.10 - 1.21) |
| Quartiles |  |  |  |  |
| Q1 | Reference | Reference | Reference | Reference |
| Q2 | 1.29 (1.10 - 1.52) | 1.20 (1.02 - 1.41) | 1.16 (0.99 - 1.36) | 1.15 (0.97 - 1.35) |
| Q3 | 1.47 (1.26 - 1.72) | 1.31 (1.11 - 1.53) | 1.23 (1.04 - 1.44) | 1.20 (1.02 - 1.41) |
| Q4 | 1.94 (1.67 - 2.25) | 1.68 (1.43 - 1.97) | 1.53 (1.30 - 1.80) | 1.48 (1.25 - 1.74) |
| P for trend | <0.001 | <0.001 | <0.001 | <0.001 |
| **TyG** |  |  |  |  |
| Per SD increase | 1.36 (1.29 - 1.42) | 1.23 (1.17 - 1.30) | 1.18 (1.11 - 1.25) | 1.18 (1.11 - 1.26) |
| Quartiles |  |  |  |  |
| Q1 | Reference | Reference | Reference | Reference |
| Q2 | 1.40 (1.19 - 1.65) | 1.30 (1.10 - 1.54) | 1.25 (1.06 - 1.48) | 1.24 (1.05 - 1.46) |
| Q3 | 1.63 (1.39 - 1.92) | 1.46 (1.24 - 1.71) | 1.36 (1.16 - 1.61) | 1.34 (1.13 - 1.58) |
| Q4 | 2.17 (1.87 - 2.53) | 1.83 (1.56 - 2.13) | 1.67 (1.42 - 1.96) | 1.58 (1.33 - 1.87) |
| P for trend | <0.001 | <0.001 | <0.001 | <0.001 |
| **TyG-WC** |  |  |  |  |
| Per SD increase | 1.48 (1.38 - 1.58) | 1.38 (1.29 - 1.48) | 1.31 (1.22 - 1.41) | 1.31 (1.22 - 1.41) |
| Quartiles |  |  |  |  |
| Q1 | Reference | Reference | Reference | Reference |
| Q2 | 1.36 (1.13 - 1.63) | 1.29 (1.08 - 1.55) | 1.26 (1.05 - 1.52) | 1.26 (1.05 - 1.52) |
| Q3 | 2.22 (1.88 - 2.62) | 2.05 (1.73 - 2.42) | 1.95 (1.64 - 2.31) | 1.94 (1.64 - 2.30) |
| Q4 | 3.07 (2.61 - 3.60) | 2.72 (2.31 - 3.20) | 2.46 (2.07 - 2.92) | 2.46 (2.07 - 2.92) |
| P for trend | <0.001 | <0.001 | <0.001 | <0.001 |
| **TyG-BMI** |  |  |  |  |
| Per SD increase | 1.53 (1.46 - 1.60) | 1.53 (1.45 - 1.60) | 1.44 (1.36 - 1.53) | 1.45 (1.37 - 1.54) |
| Quartiles |  |  |  |  |
| Q1 | Reference | Reference | Reference | Reference |
| Q2 | 1.56 (1.28 - 1.90) | 1.42 (1.16 - 1.73) | 1.34 (1.10 - 1.64) | 1.34 (1.09 - 1.64) |
| Q3 | 2.76 (2.30 - 3.30) | 2.56 (2.13 - 3.08) | 2.33 (1.93 - 2.82) | 2.34 (1.93 - 2.83) |
| Q4 | 4.37 (3.68 - 5.19) | 4.25 (3.56 - 5.07) | 3.74 (3.09 - 4.54) | 3.78 (3.11 - 4.58) |
| P for trend | <0.001 | <0.001 | <0.001 | <0.001 |
| **METS-IR** |  |  |  |  |
| Per SD increase | 1.55 (1.48 - 1.63) | 1.55 (1.47 - 1.63) | 1.50 (1.41 - 1.60) | 1.51 (1.42 - 1.61) |
| Quartiles |  |  |  |  |
| Q1 | Reference | Reference | Reference | Reference |
| Q2 | 1.37 (1.12 - 1.69) | 1.26 (1.02 - 1.56) | 1.31 (1.05 - 1.62) | 1.31 (1.06 - 1.63) |
| Q3 | 3.13 (2.61 - 3.74) | 2.95 (2.45 - 3.56) | 3.08 (2.51 - 3.78) | 3.11 (2.53 - 3.81) |
| Q4 | 4.64 (3.90 - 5.53) | 4.55 (3.79 - 5.45) | 4.73 (3.82 - 5.86) | 4.79 (3.87 - 5.94) |
| P for trend | <0.001 | <0.001 | <0.001 | <0.001 |
| **METS-VF** |  |  |  |  |
| Per SD increase | 1.84 (1.72 - 1.98) | 1.80 (1.64 - 1.98) | 1.64 (1.50 - 1.79) | 1.64 (1.50 - 1.79) |
| Quartiles |  |  |  |  |
| Q1 | Reference | Reference | Reference | Reference |
| Q2 | 1.54 (1.24 - 1.90) | 1.53 (1.24 - 1.89) | 1.56 (1.26 - 1.93) | 1.56 (1.26 - 1.93) |
| Q3 | 3.11 (2.57 - 3.76) | 3.00 (2.47 - 3.64) | 3.03 (2.49 - 3.67) | 3.02 (2.49 - 3.67) |
| Q4 | 5.54 (4.63 - 6.64) | 4.88 (4.01 - 5.95) | 4.84 (3.97 - 5.90) | 4.88 (4.00 - 5.95) |
| P for trend | <0.001 | <0.001 | <0.001 | <0.001 |

Model 1 was unadjusted;

Model 2 was adjusted for age, sex, BMI, current smoking, current drinking, SBP, and DBP;

Model 3 was further adjusted for ALT, AST, TG, HDL-C, eGFR, UA, and FPG;

Model 4 was additionally adjusted for DM, CHD, dyslipidemia, hyperuricemia, lipid-lowering drugs, antidiabetic agents, diuretics, beta-blockers, calcium channel blockers, and ACEI/ARBs.

Table S17. Sensitivity analysis of the associations between metabolic indices and multiple SRCs was performed after excluding participants with nephrolithiasis.

| Variables | Model 1 | Model 2 | Model 3 | Model 4 |
| --- | --- | --- | --- | --- |
|  | OR (95% CI) | OR (95% CI) | OR (95% CI) | OR (95% CI) |
| **TG/HDL-C** |  |  |  |  |
| Per SD increase | 1.31 (1.27 - 1.36) | 1.24 (1.20 - 1.29) | 1.20 (1.16 - 1.25) | 1.20 (1.16 - 1.25) |
| Quartiles |  |  |  |  |
| Q1 | Reference | Reference | Reference | Reference |
| Q2 | 1.50 (1.27 - 1.77) | 1.23 (1.04 - 1.46) | 1.15 (0.97 - 1.37) | 1.15 (0.97 - 1.37) |
| Q3 | 2.17 (1.85 - 2.54) | 1.59 (1.35 - 1.87) | 1.40 (1.18 - 1.66) | 1.39 (1.17 - 1.65) |
| Q4 | 3.26 (2.81 - 3.78) | 2.29 (1.95 - 2.68) | 1.78 (1.46 - 2.18) | 1.77 (1.44 - 2.16) |
| P for trend | <0.001 | <0.001 | <0.001 | <0.001 |
| **TyG** |  |  |  |  |
| Per SD increase | 1.54 (1.47 - 1.61) | 1.37 (1.31 - 1.44) | 1.26 (1.19 - 1.33) | 1.24 (1.17 - 1.32) |
| Quartiles |  |  |  |  |
| Q1 | Reference | Reference | Reference | Reference |
| Q2 | 1.63 (1.38 - 1.93) | 1.39 (1.17 - 1.65) | 1.33 (1.12 - 1.58) | 1.32 (1.11 - 1.57) |
| Q3 | 2.41 (2.05 - 2.82) | 1.84 (1.56 - 2.17) | 1.71 (1.45 - 2.02) | 1.69 (1.43 - 2.00) |
| Q4 | 3.45 (2.96 - 4.02) | 2.35 (2.01 - 2.76) | 2.03 (1.71 - 2.41) | 1.99 (1.68 - 2.37) |
| P for trend | <0.001 | <0.001 | <0.001 | <0.001 |
| **TyG-WC** |  |  |  |  |
| Per SD increase | 1.71 (1.61 - 1.82) | 1.58 (1.49 - 1.69) | 1.48 (1.39 - 1.58) | 1.47 (1.38 - 1.57) |
| Quartiles |  |  |  |  |
| Q1 | Reference | Reference | Reference | Reference |
| Q2 | 1.35 (1.14 - 1.60) | 1.28 (1.07 - 1.52) | 1.25 (1.05 - 1.49) | 1.25 (1.05 - 1.49) |
| Q3 | 2.09 (1.78 - 2.45) | 1.90 (1.62 - 2.24) | 1.83 (1.55 - 2.15) | 1.81 (1.54 - 2.14) |
| Q4 | 3.63 (3.12 - 4.21) | 3.18 (2.73 - 3.70) | 2.95 (2.53 - 3.45) | 2.92 (2.50 - 3.41) |
| P for trend | <0.001 | <0.001 | <0.001 | <0.001 |
| **TyG-BMI** |  |  |  |  |
| Per SD increase | 1.72 (1.65 - 1.79) | 1.71 (1.63 - 1.79) | 1.59 (1.51 - 1.67) | 1.59 (1.51 - 1.67) |
| Quartiles |  |  |  |  |
| Q1 | Reference | Reference | Reference | Reference |
| Q2 | 1.54 (1.25 - 1.90) | 1.36 (1.10 - 1.68) | 1.23 (0.99 - 1.52) | 1.22 (0.98 - 1.50) |
| Q3 | 4.18 (3.49 - 5.01) | 3.82 (3.18 - 4.60) | 3.22 (2.66 - 3.90) | 3.20 (2.64 - 3.88) |
| Q4 | 6.34 (5.32 - 7.56) | 6.16 (5.14 - 7.38) | 4.90 (4.03 - 5.95) | 4.88 (4.01 - 5.93) |
| P for trend | <0.001 | <0.001 | <0.001 | <0.001 |
| **METS-IR** |  |  |  |  |
| Per SD increase | 1.78 (1.71 - 1.86) | 1.77 (1.69 - 1.85) | 1.66 (1.57 - 1.75) | 1.66 (1.57 - 1.76) |
| Quartiles |  |  |  |  |
| Q1 | Reference | Reference | Reference | Reference |
| Q2 | 1.75 (1.40 - 2.18) | 1.53 (1.23 - 1.92) | 1.49 (1.19 - 1.86) | 1.48 (1.18 - 1.85) |
| Q3 | 4.40 (3.62 - 5.36) | 4.02 (3.29 - 4.91) | 3.80 (3.10 - 4.66) | 3.79 (3.09 - 4.64) |
| Q4 | 8.56 (7.10 - 10.33) | 8.16 (6.72 - 9.91) | 7.54 (6.17 - 9.21) | 7.53 (6.16 - 9.20) |
| P for trend | <0.001 | <0.001 | <0.001 | <0.001 |
| **METS-VF** |  |  |  |  |
| Per SD increase | 2.45 (2.28 - 2.63) | 1.90 (1.74 - 2.08) | 2.09 (1.88 - 2.31) | 2.09 (1.89 - 2.32) |
| Quartiles |  |  |  |  |
| Q1 | Reference | Reference | Reference | Reference |
| Q2 | 1.46 (1.16 - 1.83) | 1.45 (1.15 - 1.82) | 1.49 (1.18 - 1.88) | 1.49 (1.18 - 1.88) |
| Q3 | 3.98 (3.26 - 4.86) | 3.76 (3.07 - 4.60) | 3.87 (3.16 - 4.74) | 3.87 (3.16 - 4.74) |
| Q4 | 9.39 (7.78 - 11.33) | 7.33 (5.97 - 9.01) | 7.67 (6.24 - 9.43) | 7.65 (6.22 - 9.41) |
| P for trend | <0.001 | <0.001 | <0.001 | <0.001 |

Model 1 was unadjusted;

Model 2 was adjusted for age, sex, BMI, current smoking, current drinking, SBP, and DBP;

Model 3 was further adjusted for ALT, AST, TG, HDL-C, eGFR, UA, and FPG;

Model 4 was additionally adjusted for DM, CHD, dyslipidemia, hyperuricemia, lipid-lowering drugs, antidiabetic agents, diuretics, beta-blockers, calcium channel blockers, and ACEI/ARBs.

Table S18. Sensitivity analysis of the associations between metabolic indices and SRCs was performed after excluding participants with hyperuricemia.

| Variables | Model 1 | Model 2 | Model 3 | Model 4 |
| --- | --- | --- | --- | --- |
|  | OR (95% CI) | OR (95% CI) | OR (95% CI) | OR (95% CI) |
| **TG/HDL-C** |  |  |  |  |
| Per SD increase | 1.21 (1.17 - 1.24) | 1.12 (1.08 - 1.15) | 1.11 (1.08 - 1.15) | 1.10 (1.07 - 1.14) |
| Quartiles |  |  |  |  |
| Q1 | Reference | Reference | Reference | Reference |
| Q2 | 1.18 (1.10 - 1.28) | 1.06 (0.98 - 1.15) | 1.07 (0.99 - 1.16) | 1.06 (0.98 - 1.15) |
| Q3 | 1.31 (1.22 - 1.42) | 1.09 (1.01 - 1.18) | 1.11 (1.03 - 1.21) | 1.09 (1.01 - 1.19) |
| Q4 | 1.63 (1.51 - 1.77) | 1.25 (1.15 - 1.36) | 1.28 (1.17 - 1.39) | 1.26 (1.15 - 1.37) |
| P for trend | <0.001 | <0.001 | <0.001 | <0.001 |
| **TyG** |  |  |  |  |
| Per SD increase | 1.25 (1.22 - 1.29) | 1.16 (1.13 - 1.19) | 1.15 (1.12 - 1.19) | 1.13 (1.09 - 1.17) |
| Quartiles |  |  |  |  |
| Q1 | Reference | Reference | Reference | Reference |
| Q2 | 1.21 (1.12 - 1.31) | 1.14 (1.05 - 1.23) | 1.14 (1.05 - 1.23) | 1.12 (1.04 - 1.22) |
| Q3 | 1.28 (1.18 - 1.38) | 1.15 (1.06 - 1.24) | 1.15 (1.06 - 1.25) | 1.13 (1.04 - 1.23) |
| Q4 | 1.69 (1.57 - 1.83) | 1.37 (1.26 - 1.48) | 1.36 (1.26 - 1.48) | 1.35 (1.24 - 1.47) |
| P for trend | <0.001 | <0.001 | <0.001 | <0.001 |
| **TyG-WC** |  |  |  |  |
| Per SD increase | 1.33 (1.29 - 1.39) | 1.25 (1.20 - 1.30) | 1.25 (1.20 - 1.30) | 1.24 (1.19 - 1.29) |
| Quartiles |  |  |  |  |
| Q1 | Reference | Reference | Reference | Reference |
| Q2 | 1.26 (1.16 - 1.36) | 1.21 (1.12 - 1.32) | 1.21 (1.11 - 1.31) | 1.21 (1.11 - 1.31) |
| Q3 | 1.38 (1.28 - 1.50) | 1.28 (1.18 - 1.39) | 1.27 (1.17 - 1.38) | 1.26 (1.16 - 1.37) |
| Q4 | 1.71 (1.58 - 1.85) | 1.53 (1.41 - 1.66) | 1.49 (1.38 - 1.62) | 1.48 (1.36 - 1.61) |
| P for trend | <0.001 | <0.001 | <0.001 | <0.001 |
| **TyG-BMI** |  |  |  |  |
| Per SD increase | 1.34 (1.31 - 1.38) | 1.30 (1.26 - 1.33) | 1.27 (1.22 - 1.31) | 1.27 (1.22 - 1.31) |
| Quartiles |  |  |  |  |
| Q1 | Reference | Reference | Reference | Reference |
| Q2 | 1.32 (1.22 - 1.43) | 1.17 (1.07 - 1.29) | 1.14 (1.03 - 1.26) | 1.13 (1.02 - 1.24) |
| Q3 | 1.76 (1.63 - 1.91) | 1.58 (1.42 - 1.75) | 1.50 (1.32 - 1.70) | 1.48 (1.31 - 1.68) |
| Q4 | 2.34 (2.16 - 2.53) | 2.20 (1.90 - 2.55) | 2.03 (1.68 - 2.45) | 2.01 (1.66 - 2.43) |
| P for trend | <0.001 | <0.001 | <0.001 | <0.001 |
| **METS-IR** |  |  |  |  |
| Per SD increase | 1.36 (1.33 - 1.40) | 1.30 (1.26 - 1.34) | 1.30 (1.25 - 1.35) | 1.30 (1.25 - 1.35) |
| Quartiles |  |  |  |  |
| Q1 | Reference | Reference | Reference | Reference |
| Q2 | 1.43 (1.32 - 1.55) | 1.34 (1.22 - 1.46) | 1.29 (1.18 - 1.42) | 1.27 (1.16 - 1.40) |
| Q3 | 1.80 (1.66 - 1.95) | 1.68 (1.51 - 1.86) | 1.60 (1.44 - 1.77) | 1.56 (1.40 - 1.74) |
| Q4 | 2.47 (2.28 - 2.68) | 2.37 (2.07 - 2.71) | 2.22 (1.93 - 2.55) | 2.15 (1.86 - 2.48) |
| P for trend | <0.001 | <0.001 | <0.001 | <0.001 |
| **METS-VF** |  |  |  |  |
| Per SD increase | 1.55 (1.49 - 1.60) | 1.49 (1.43 - 1.56) | 1.49 (1.43 - 1.56) | 1.47 (1.41 - 1.54) |
| Quartiles |  |  |  |  |
| Q1 | Reference | Reference | Reference | Reference |
| Q2 | 1.45 (1.33 - 1.58) | 1.58 (1.45 - 1.73) | 1.59 (1.45 - 1.74) | 1.59 (1.45 - 1.74) |
| Q3 | 1.83 (1.68 - 1.99) | 1.94 (1.77 - 2.12) | 1.94 (1.77 - 2.12) | 1.92 (1.75 - 2.10) |
| Q4 | 2.87 (2.64 - 3.11) | 2.61 (2.38 - 2.86) | 2.62 (2.38 - 2.87) | 2.54 (2.31 - 2.79) |
| P for trend | <0.001 | <0.001 | <0.001 | <0.001 |

Model 1 was unadjusted;

Model 2 was adjusted for age, sex, BMI, current smoking, current drinking, SBP, DBP;

Model 3 was further adjusted for ALT, AST, TG, HDL-C, eGFR, UA, FPG;

Model 4 was additionally adjusted for DM, dyslipidemia, CHD, nephrolithiasis, lipid-lowering drugs, antidiabetic agents, diuretics, beta-blockers, calcium channel blockers, and ACEI/ARBs.

Abbreviations: WC, waist circumference; BMI, body mass index; TyG, triglyceride-glucose; METS-IR, metabolic score for insulin resistance; METS-VF, metabolic score for visceral fat; OR, odds ratio; CI, confidence interval; Other abbreviations, see Table 1.

Table S19. Sensitivity analysis of the associations between metabolic indices and large SRCs was performed after excluding participants with hyperuricemia.

| Variables | Model 1 | Model 2 | Model 3 | Model 4 |
| --- | --- | --- | --- | --- |
|  | OR (95% CI) | OR (95% CI) | OR (95% CI) | OR (95% CI) |
| **TG/HDL-C** |  |  |  |  |
| Per SD increase | 1.27 (1.21 - 1.32) | 1.21 (1.15 - 1.27) | 1.20 (1.14 - 1.26) | 1.19 (1.13 - 1.26) |
| Quartiles |  |  |  |  |
| Q1 | Reference | Reference | Reference | Reference |
| Q2 | 1.38 (1.17 - 1.63) | 1.18 (0.99 - 1.39) | 1.16 (0.98 - 1.37) | 1.16 (0.98 - 1.37) |
| Q3 | 1.58 (1.34 - 1.86) | 1.24 (1.04 - 1.47) | 1.21 (1.02 - 1.44) | 1.20 (1.01 - 1.42) |
| Q4 | 1.86 (1.57 - 2.19) | 1.37 (1.15 - 1.63) | 1.32 (1.11 - 1.58) | 1.29 (1.08 - 1.55) |
| P for trend | <0.001 | <0.001 | <0.001 | <0.001 |
| **TyG** |  |  |  |  |
| Per SD increase | 1.35 (1.28 - 1.42) | 1.27 (1.20 - 1.34) | 1.25 (1.18 - 1.32) | 1.24 (1.17 - 1.32) |
| Quartiles |  |  |  |  |
| Q1 | Reference | Reference | Reference | Reference |
| Q2 | 1.38 (1.17 - 1.64) | 1.29 (1.09 - 1.52) | 1.25 (1.05 - 1.48) | 1.24 (1.05 - 1.47) |
| Q3 | 1.53 (1.29 - 1.81) | 1.36 (1.15 - 1.61) | 1.30 (1.10 - 1.55) | 1.28 (1.08 - 1.53) |
| Q4 | 2.04 (1.74 - 2.41) | 1.70 (1.44 - 2.01) | 1.60 (1.35 - 1.89) | 1.53 (1.27 - 1.83) |
| P for trend | <0.001 | <0.001 | <0.001 | <0.001 |
| **TyG-WC** |  |  |  |  |
| Per SD increase | 1.41 (1.31 - 1.52) | 1.31 (1.22 - 1.42) | 1.30 (1.21 - 1.40) | 1.29 (1.19 - 1.39) |
| Quartiles |  |  |  |  |
| Q1 | Reference | Reference | Reference | Reference |
| Q2 | 1.83 (1.49 - 2.24) | 1.76 (1.44 - 2.16) | 1.76 (1.44 - 2.16) | 1.77 (1.44 - 2.17) |
| Q3 | 2.70 (2.23 - 3.27) | 2.52 (2.07 - 3.05) | 2.50 (2.06 - 3.03) | 2.50 (2.06 - 3.04) |
| Q4 | 3.93 (3.27 - 4.73) | 3.48 (2.89 - 4.19) | 3.44 (2.85 - 4.14) | 3.43 (2.84 - 4.14) |
| P for trend | <0.001 | <0.001 | <0.001 | <0.001 |
| **TyG-BMI** |  |  |  |  |
| Per SD increase | 1.50 (1.43 - 1.58) | 1.49 (1.41 - 1.57) | 1.44 (1.36 - 1.53) | 1.46 (1.37 - 1.54) |
| Quartiles |  |  |  |  |
| Q1 | Reference | Reference | Reference | Reference |
| Q2 | 1.82 (1.49 - 2.21) | 1.65 (1.35 - 2.02) | 1.61 (1.32 - 1.98) | 1.61 (1.32 - 1.98) |
| Q3 | 2.82 (2.33 - 3.40) | 2.57 (2.12 - 3.12) | 2.47 (2.03 - 3.01) | 2.50 (2.05 - 3.04) |
| Q4 | 4.23 (3.53 - 5.07) | 4.01 (3.33 - 4.83) | 3.78 (3.11 - 4.59) | 3.85 (3.16 - 4.68) |
| P for trend | <0.001 | <0.001 | <0.001 | <0.001 |
| **METS-IR** |  |  |  |  |
| Per SD increase | 1.53 (1.45 - 1.61) | 1.52 (1.43 - 1.60) | 1.48 (1.38 - 1.58) | 1.48 (1.39 - 1.58) |
| Quartiles |  |  |  |  |
| Q1 | Reference | Reference | Reference | Reference |
| Q2 | 1.73 (1.41 - 2.12) | 1.58 (1.29 - 1.95) | 1.62 (1.30 - 2.01) | 1.56 (1.26 - 1.92) |
| Q3 | 3.13 (2.59 - 3.78) | 2.90 (2.39 - 3.53) | 2.98 (2.41 - 3.69) | 2.83 (2.32 - 3.46) |
| Q4 | 4.40 (3.66 - 5.29) | 4.20 (3.47 - 5.09) | 4.31 (3.45 - 5.39) | 4.04 (3.29 - 4.96) |
| P for trend | <0.001 | <0.001 | <0.001 | <0.001 |
| **METS-VF** |  |  |  |  |
| Per SD increase | 1.73 (1.60 - 1.87) | 1.62 (1.48 - 1.78) | 1.66 (1.52 - 1.83) | 1.66 (1.51 - 1.82) |
| Quartiles |  |  |  |  |
| Q1 | Reference | Reference | Reference | Reference |
| Q2 | 1.50 (1.19 - 1.89) | 1.70 (1.35 - 2.15) | 1.71 (1.35 - 2.16) | 1.70 (1.35 - 2.15) |
| Q3 | 2.91 (2.36 - 3.59) | 3.29 (2.66 - 4.08) | 3.33 (2.69 - 4.13) | 3.31 (2.68 - 4.11) |
| Q4 | 5.13 (4.21 - 6.25) | 5.33 (4.29 - 6.63) | 5.51 (4.43 - 6.86) | 5.48 (4.41 - 6.82) |
| P for trend | <0.001 | <0.001 | <0.001 | <0.001 |

Model 1 was unadjusted;

Model 2 was adjusted for age, sex, BMI, current smoking, current drinking, SBP, DBP;

Model 3 was further adjusted for ALT, AST, TG, HDL-C, eGFR, UA, FPG;

Model 4 was additionally adjusted for DM, dyslipidemia, CHD, nephrolithiasis, lipid-lowering drugs, antidiabetic agents, diuretics, beta-blockers, calcium channel blockers, and ACEI/ARBs.

Abbreviations: WC, waist circumference; BMI, body mass index; TyG, triglyceride-glucose; METS-IR, metabolic score for insulin resistance; METS-VF, metabolic score for visceral fat; OR, odds ratio; CI, confidence interval; Other abbreviations, see Table 1.

Table S20. Sensitivity analysis of the associations between metabolic indices and multiple SRCs was performed after excluding participants with hyperuricemia.

| Variables | Model 1 | Model 2 | Model 3 | Model 4 |
| --- | --- | --- | --- | --- |
|  | OR (95% CI) | OR (95% CI) | OR (95% CI) | OR (95% CI) |
| **TG/HDL-C** |  |  |  |  |
| Per SD increase | 1.36 (1.31 - 1.41) | 1.29 (1.23 - 1.34) | 1.23 (1.18 - 1.29) | 1.24 (1.18 - 1.29) |
| Quartiles |  |  |  |  |
| Q1 | Reference | Reference | Reference | Reference |
| Q2 | 1.38 (1.17 - 1.62) | 1.16 (0.98 - 1.37) | 1.10 (0.93 - 1.30) | 1.11 (0.94 - 1.31) |
| Q3 | 2.09 (1.79 - 2.44) | 1.61 (1.37 - 1.89) | 1.48 (1.26 - 1.74) | 1.50 (1.27 - 1.76) |
| Q4 | 3.16 (2.73 - 3.67) | 2.32 (1.98 - 2.72) | 2.02 (1.71 - 2.38) | 2.04 (1.73 - 2.41) |
| P for trend | <0.001 | <0.001 | <0.001 | <0.001 |
| **TyG** |  |  |  |  |
| Per SD increase | 1.56 (1.49 - 1.63) | 1.40 (1.33 - 1.47) | 1.27 (1.19 - 1.34) | 1.27 (1.20 - 1.35) |
| Quartiles |  |  |  |  |
| Q1 | Reference | Reference | Reference | Reference |
| Q2 | 1.90 (1.61 - 2.25) | 1.64 (1.39 - 1.95) | 1.49 (1.26 - 1.77) | 1.49 (1.25 - 1.77) |
| Q3 | 2.53 (2.15 - 2.99) | 2.00 (1.69 - 2.37) | 1.70 (1.44 - 2.02) | 1.71 (1.44 - 2.03) |
| Q4 | 3.88 (3.31 - 4.54) | 2.73 (2.32 - 3.21) | 2.04 (1.71 - 2.44) | 2.05 (1.71 - 2.46) |
| P for trend | <0.001 | <0.001 | <0.001 | <0.001 |
| **TyG-WC** |  |  |  |  |
| Per SD increase | 1.58 (1.48 - 1.69) | 1.47 (1.37 - 1.57) | 1.34 (1.25 - 1.44) | 1.34 (1.25 - 1.44) |
| Quartiles |  |  |  |  |
| Q1 | Reference | Reference | Reference | Reference |
| Q2 | 1.59 (1.33 - 1.91) | 1.53 (1.28 - 1.83) | 1.48 (1.24 - 1.78) | 1.48 (1.24 - 1.78) |
| Q3 | 2.41 (2.04 - 2.85) | 2.23 (1.88 - 2.65) | 2.10 (1.77 - 2.49) | 2.09 (1.76 - 2.48) |
| Q4 | 4.03 (3.43 - 4.72) | 3.54 (3.01 - 4.16) | 3.09 (2.62 - 3.64) | 3.08 (2.61 - 3.63) |
| P for trend | <0.001 | <0.001 | <0.001 | <0.001 |
| **TyG-BMI** |  |  |  |  |
| Per SD increase | 1.66 (1.59 - 1.74) | 1.64 (1.57 - 1.72) | 1.49 (1.41 - 1.57) | 1.49 (1.41 - 1.58) |
| Quartiles |  |  |  |  |
| Q1 | Reference | Reference | Reference | Reference |
| Q2 | 1.48 (1.23 - 1.78) | 1.31 (1.09 - 1.59) | 1.16 (0.95 - 1.40) | 1.15 (0.95 - 1.40) |
| Q3 | 3.27 (2.76 - 3.87) | 2.96 (2.49 - 3.52) | 2.41 (2.01 - 2.88) | 2.41 (2.01 - 2.88) |
| Q4 | 5.01 (4.26 - 5.89) | 4.74 (4.00 - 5.60) | 3.56 (2.96 - 4.27) | 3.57 (2.97 - 4.29) |
| P for trend | <0.001 | <0.001 | <0.001 | <0.001 |
| **METS-IR** |  |  |  |  |
| Per SD increase | 1.74 (1.66 - 1.82) | 1.72 (1.63 - 1.80) | 1.55 (1.46 - 1.65) | 1.56 (1.47 - 1.66) |
| Quartiles |  |  |  |  |
| Q1 | Reference | Reference | Reference | Reference |
| Q2 | 1.54 (1.26 - 1.87) | 1.36 (1.12 - 1.66) | 1.28 (1.04 - 1.57) | 1.28 (1.04 - 1.57) |
| Q3 | 3.10 (2.59 - 3.70) | 2.81 (2.34 - 3.38) | 2.52 (2.07 - 3.08) | 2.53 (2.07 - 3.09) |
| Q4 | 6.37 (5.39 - 7.53) | 5.99 (5.04 - 7.13) | 5.07 (4.13 - 6.22) | 5.10 (4.15 - 6.27) |
| P for trend | <0.001 | <0.001 | <0.001 | <0.001 |
| **METS-VF** |  |  |  |  |
| Per SD increase | 2.45 (2.28 - 2.63) | 1.90 (1.74 - 2.08) | 2.09 (1.88 - 2.31) | 2.09 (1.89 - 2.32) |
| Quartiles |  |  |  |  |
| Q1 | Reference | Reference | Reference | Reference |
| Q2 | 1.43 (1.14 - 1.79) | 1.43 (1.14 - 1.80) | 1.48 (1.17 - 1.86) | 1.48 (1.17 - 1.86) |
| Q3 | 3.37 (2.76 - 4.11) | 3.27 (2.67 - 4.00) | 3.34 (2.72 - 4.09) | 3.33 (2.72 - 4.09) |
| Q4 | 7.55 (6.25 - 9.11) | 6.48 (5.33 - 7.89) | 6.43 (5.23 - 7.91) | 6.41 (5.21 - 7.88) |
| P for trend | <0.001 | <0.001 | <0.001 | <0.001 |

Model 1 was unadjusted;

Model 2 was adjusted for age, sex, BMI, current smoking, current drinking, SBP, DBP;

Model 3 was further adjusted for ALT, AST, TG, HDL-C, eGFR, UA, FPG;

Model 4 was additionally adjusted for DM, dyslipidemia, CHD, nephrolithiasis, lipid-lowering drugs, antidiabetic agents, diuretics, beta-blockers, calcium channel blockers, and ACEI/ARBs.

Abbreviations: WC, waist circumference; BMI, body mass index; TyG, triglyceride-glucose; METS-IR, metabolic score for insulin resistance; METS-VF, metabolic score for visceral fat; OR, odds ratio; CI, confidence interval; Other abbreviations, see Table 1.
